# Inclusion and exclusion criteria used in non-specific low back pain trials: a review of randomised controlled trials published between 2006 and 2012

**DOI:** 10.1186/s12891-018-2034-6

**Published:** 2018-04-12

**Authors:** Pål André Amundsen, David W. Evans, Dévan Rajendran, Philip Bright, Tom Bjørkli, Sandra Eldridge, Rachelle Buchbinder, Martin Underwood, Robert Froud

**Affiliations:** 1 Institute of Health Sciences, Kristiania University College, Prinsens Gate 7-9, 0152 Oslo, Norway; 2 Warwick Clinical Trials Unit. Warwick Medical School, University of Warwick, Gibbet Hill Road, Coventry, CV4 7AL UK; 3 Centre of Precision Rehabilitation for Spinal Pain, School of Sport, Exercise and Rehabilitation Sciences, University of Birmingham, Edgbaston, Birmingham, B15 2TT UK; 4 European School of Osteopathy, The Street, Boxley, Maidstone, Kent, ME14 3DZ UK; 5 Monash Department of Clinical Epidemiology, Cabrini Institute and Department of Epidemiology and Preventive Medicine, Monash University, Suite 41, Cabrini Medical Centre, 183 Wattletree Road, Malvern, Melbourne, Victoria 3144 Australia; 6 Centre for Primary Care and Public Health, Queen Mary University of London, 58 Turner Street, Whitechapel, London, E1 2AB UK

**Keywords:** Low back pain, Non-specific, Inclusion criteria, Exclusion criteria, Systematic review, Definitions

## Abstract

**Background:**

Low back pain is a common health complaint resulting in substantial economic burden. Each year, upwards of 20 randomised controlled trials (RCTs) evaluating interventions for non-specific low back pain are published. Use of the term non-specific low back pain has been criticised on the grounds of encouraging heterogeneity and hampering interpretation of findings due to possible heterogeneous causes, challenging meta-analyses. We explored selection criteria used in trials of treatments for nsLBP.

**Methods:**

A systematic review of English-language reports of RCTs in nsLBP population samples, published between 2006 and 2012, identified from MEDLINE, EMBASE, and the Cochrane Library databases, using a mixed-methods approach to analysis. Study inclusion and exclusion criteria were extracted, thematically categorised, and then descriptive statistics were used to summarise the prevalence by emerging category.

**Results:**

We included 168 studies. Two inclusion themes (anatomical area, and symptoms and signs) were identified. Anatomical area was most reported as between costal margins and gluteal folds (*n* = 8, 5%), while low back pain (*n* = 150, 89%) with or without referred leg pain (*n* = 27, 16%) was the most reported symptom. Exclusion criteria comprised 21 themes. Previous or scheduled surgery (*n* = 84, 50%), pregnancy (*n* = 81, 48%), malignancy (*n* = 78, 46%), trauma (*n* = 63, 37%) and psychological conditions (*n* = 58, 34%) were the most common. Sub-themes of exclusion criteria mostly related to neurological signs and symptoms: nerve root compromise (*n* = 44, 26%), neurological signs (*n* = 34, 20%) or disc herniation (*n* = 30, 18%). Specific conditions that were most often exclusion criteria were spondylolisthesis (*n* = 35, 21%), spinal stenosis (*n* = 31, 18%) or osteoporosis (*n* = 27, 16%).

**Conclusion:**

RCTs of interventions for non-specific low back pain have incorporated diverse inclusion and exclusion criteria. Guidance on standardisation of inclusion and exclusion criteria for nsLBP trials will increase clinical homogeneity, facilitating greater interpretation of between-trial comparisons and meta-analyses. We propose a template for reporting inclusion and exclusion criteria.

**Electronic supplementary material:**

The online version of this article (10.1186/s12891-018-2034-6) contains supplementary material, which is available to authorized users.

## Background

Low back pain (LBP) is a common and costly problem resulting in a substantial personal, social and economic burden globally [1, 2]. Low back and neck pain are ranked fourth in terms of disability-adjusted life years, and the leading cause of activity limitation and work absence globally [2–4]. The lifetime prevalence of LBP is between 60 and 84% [5, 6]. Most episodes of LBP are self-limiting and not related to serious disease [5, 7]. A specific cause of LBP is currently identifiable in only a small minority of people (5–15%) and includes serious pathology such as malignancy, vertebral fracture, infection or axial spondyloarthritis [7–9]. The term ‘non-specific’ LBP (nsLBP) is used to refer to instances where no specific cause has been identified [7, 8, 10–13]. The term has no agreed definition despite being used by organisations such as the World Health Organization, International Association for the Study of Pain, Backpain Europe, and the (UK) National Institute for Health and Care Excellence [7, 9, 14–22].

The annual rate of publication for randomised controlled trials (RCTs) that test interventions for people with nsLBP has increased from an average of 5.3 RCTs per year between 1980 and 1999, to 23.3 per year between 2000 and 2012 [23]. Interpretation of the results of these numerous RCTs, requires a good understanding of the study populations that have been included. Similarly, to synthesise the results of RCTs in meta-analyses requires study populations to be reasonably homogeneous across trials. Study populations are determined by the selection or eligibility (inclusion and exclusion) criteria that form the framework for sampling [24]. Little research has examined inclusion and exclusion criteria used in RCTs of LBP populations [24–26]; studies that did, found ambiguous identification of neurological involvement in the selection criteria, and inconsistencies across clinical decision guidelines for LBP. The extent to which trialists have used a consistent approach to identifying people with nsLBP is currently unclear.

As part of a larger study systematically reviewing RCTs of treatment for non-specific LBP, the aims relevant to this paper were to systematically describe the inclusion and exclusion criteria reported in RCTs that test interventions for nsLBP, the frequency of reporting criteria and to classify criteria by theme [23].

## Methods

Research methods in low back pain research have developed over recent decades. To assess current practice in trials, we searched for all trials of nsLBP in MEDLINE, EMBASE, and the Cochrane Register of Controlled Trials, published between January 1, 2006 and January 1, 2012. An example search strategy is included as an additional file [see Additional file 1].

Two of three reviewers (PB, DR or TB), working independently, identified all candidate RCT reports by combining all database hits in an Endnote (Version 14; Thomson Reuters, Philadelphia) library, removing duplicates, and short-listing by title and abstract. Full-texts were obtained if the titles and abstract alone contained insufficient information for assessment against the criteria (Table 1). Reports that self-identified as pilot/feasibility studies were excluded as these are by definition not set up to explore effectiveness. Additionally, the inclusion criteria might be different because the aims may be different [27]. Further, the inherent problem with low power due to the small sample sizes used, may not be able to be overcome using meta-analytical techniques due to the assumptions about underlying distributions being unrealistic [28].

**Table 1:** Inclusion and exclusion criteria and the order of their evaluation

Inclusion criterion	
RCTs of nsLBP not failing one of the eight exclusion criteria	
Order	Exclusion criteria
1	Non-English language reports
2	Studies that were not RCTs or presented insufficient information for us to determine whether randomisation was used to allocate participants
3	Reports that self-identified as pilot/feasibility studies
4	Cross-over designs (because of limited utility in the LBP field)
5	RCTs with mixed samples (e.g. neck or thoracic pain in addition to LBP), samples of participants with radiating leg pain, or referred pain extending past the knee in reports where LBP was not described as non-specific, or samples including LBP specific pathology (e.g. cancer, ankylosing spondylitis, or disc herniation) or pregnancy
6	Trials using solely objective or psychological outcome measures
7	Non-inferiority designs
8	Follow-up studies with no new outcome measures, and multiple publications. In the case of multiple publications, we included the first published article and excluded subsequent publications

RCT = Randomised controlled trial; nsLBP=Non-specific low back pain

### Data extraction and analysis

Two of three reviewers (PB, DR or TB) independently extracted data on inclusion and exclusion criteria reported in the methods section of each included trial. One reviewer (PA) entered these data into a database. Following extraction of inclusion and exclusion criteria, we used expert validation of extracted data on 20% random sample of included trials (95,1% level of agreement), as has been done in other reviews [29, 30].

To identify the categories of reported inclusion and exclusion criteria, we developed a coding framework with themes and subthemes. PA, RF and DR first familiarised themselves with the extracted data and coded each inclusion and exclusion criterion with labels that described their focus, and then grouped these coded data into clusters of similar interrelated ideas or concepts to form general categories. We used a Microsoft Excel (Microsoft, Washington) spreadsheet to generate a matrix, and the categories were then ‘charted’ into our framework matrix. We retained the terminology used within RCT reports, aiming to describe the verbatim terms used. However, decisions needed to be made during the process regarding criteria that could be conflated (e.g. spondylolisthesis grade I and spondylolisthesis grade II) to achieve useful data reduction and facilitate interpretation of results. These decisions were made through team discussion and the framework was revised and refined until all the coded data could be modelled within the structure of the framework.

We populated each theme and subtheme of our framework with frequencies of reporting. Frequency distributions were used to summarise the prevalence of reported criterion. All quantitative descriptive analyses were performed using SPSS (IBM, Washington).

## Results

**Fig. 1:**
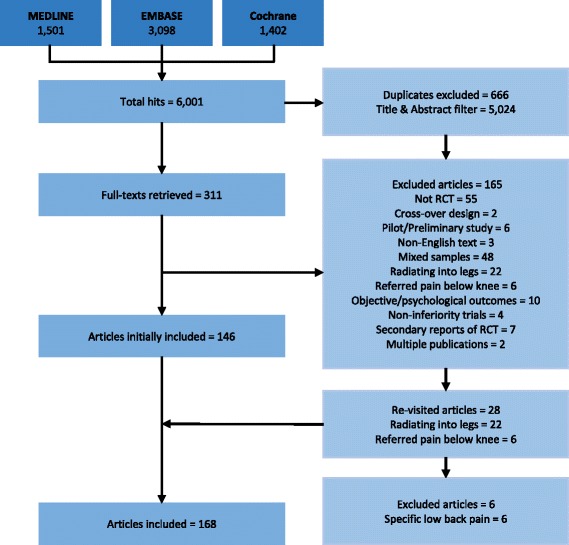
Flowchart

Our initial search identified 6001 studies; we examined full-texts of 311 of these. At full-text level, 143 articles were excluded [50–192], and 168 met the inclusion criteria [193–360] (Fig. 1) [see Additional file 2]. [see Additional file 3: Table S1] shows the characteristics of included studies, and [see Additional file 4: Table S2] shows the characteristics of excluded studies.

### Framework of inclusion and exclusion criteria

Table 2 shows our framework of themes (*n* = 3) and subthemes (*n* = 27) identified from inclusion criteria, while Table 3 shows the identified themes (*n* = 21) and subthemes (*n* = 117) from exclusion criteria.

**Table 2:** Inclusion themes and subthemes

Theme		Subthemes	n (%)
1	Anatomical area	Between costal margins and gluteal folds	8 (5)
		Below scapulae and above gluteal folds	3 (2)
		T6 or below	2 (1)
		T7 or below	2 (1)
		Low back or buttocks	2 (1)
		Between L1 and gluteal folds	2 (1)
		Between L1 and SI-joints	1 (1)
		At or above waist level	1 (1)
2	Symptoms and signs	Low back pain	150 (89)
		With or without referred Leg pain	27 (16)
		Back pain	10 (6)
		Without referred leg pain	7 (4)
		Pain exacerbated by movement	5 (3)
		Limited movement	3 (2)
		Stiffness	2 (1)
		Tension	2 (1)
		With referred leg pain	1 (1)
		Pain at rest	1 (1)
		Discomfort	1 (1)
3	Patient-reported outcome measures with score thresholds for inclusion	Visual Analogue Scale* (0 to 100)	
		≥ 40 mm	6 (4)
		≥ 30 mm	4 (2)
		≥ 65 mm	1 (1)
		≥ 35 mm	1 (1)
		≥ 20 mm	1 (1)
		≥ 10 mm	1 (1)
		Oswestry Functional Disability Index (0 to 100%)	
		≥ 30%	2 (1)
		≥ 25%	2 (1)
		≥ 20%	1 (1)
		≥ 15%	1 (1)
		Roland Morris Disability Questionnaire (0 to 24)	
		≥ 3 points	2 (1)
		≤ 4 points	1 (1)
		≤ 5 points	1 (1)
		Numeric Rating Scale (0 to 10)	
		≥ 3 points	1 (1)
		≥ 2 points	1 (1)
		Von Korff Chronic Pain Grade (0 to 4)	
		≥ Grade 1	1 (1)
		Hanover Ability Questionnaire (0 to 100%)	
		≤ 70%	1 (1)
		Short-From 36	
		“moderate pain and moderate disability (measured by adaptations of items 7 and 8 of SF-36)”	1 (1)
		Brief Pain Inventory (0 to 10 on Average pain)	
		“4 points on average pain last 6 months”	1 (1)

*Visual Analogue Scale transformed to 0 to 100 mm

**Table 3:** Exclusion themes and subthemes

Themes		Subthemes	n (%)
1	Back-related condition	Spondylolisthesis	35 (21)
		Spinal stenosis	31 (18)
		Spondylolysis	13 (8)
		Ankylosing spondylitis^a^	8 (5)
		Structural deformity	7 (4)
		Osteoporotic fracture	6 (4)
		Congenital deformation	6 (4)
		Disc disease	3 (2)
		Sacroiliitis	3 (2)
		Severe structural deformity	3 (2)
		Scoliosis	2 (1)
		Active structural deficit	2 (1)
		Severe postural abnormality	2 (1)
2	Specified, unspecified, implied systemic, rheumatologic or immunologic conditions	Inflammatory disease	37 (22)
		Osteoporosis	27 (16)
		Rheumatological disease	13 (8)
		Rheumatoid arthritis	12 (7)
		Fibromyalgia	9 (5)
		Autoimmune disease	4 (2)
		Reactive arthritis^b^	2 (1)
		Bone disease	2 (1)
		Osteoarthritis	2 (1)
		Inflammatory arthritis	1 (1)
		Arthritis	1 (1)
		Systemic Lupus Erythematosus	1 (1)
		Myofascial pain syndrome	1 (1)
		Articular impairment	1 (1)
3	Psychological	Psychiatric disorders	35 (21)
		Depression	11 (6)
		Severe psychiatric disorder	8 (5)
		Impaired cognition	7 (4)
4	Other systemic, unspecified	Systemic disease	15 (9)
		Metabolic disease	10 (6)
		Visceral disease	9 (5)
		Endocrine disorder	2 (1)
		Uterine disease	1 (1)
		Thyroid dysfunction	1 (1)
5	Trauma		63 (37)
6	Malignancy		78 (46)
7	Infectious	Infection	42 (25)
		Infectious spondylopathy	5 (3)
		Infectious disease	4 (2)
		Previous infection	1 (1)
		Abscess	1 (1)
8	Cardiovascular	Cardiac disease /insufficiency	32 (19)
		Vascular disease /insufficiency	13 (8)
		Hypertension	6 (4)
		Ischemic heart attack	2 (1)
		Claudication	2 (1)
		Aortic aneurysm	1 (1)
9	Haematological	Bleeding disorders	5 (3)
		Blood coagulation disorder	2 (1)
10	Respiratory	Respiratory disease /insufficiency	14 (8)
11	Gastrointestinal, liver or renal	Severe renal or hepatic disorder	5 (3)
		Liver disease	2 (1)
		Gastrointestinal disease	2 (1)
		Abdominal hernia	2 (1)
		Gastritis	1 (1)
		Gastric ulcer	1 (1)
		Crohn’s disease	1 (1)
		Inguinal hernia	1 (1)
12	Neurological, systemic	Myelopathy	2 (1)
		Epilepsy	2 (1)
		Seizure disorders	1 (1)
		Muscular disease	1 (1)
		Muscular dystrophy	1 (1)
13	General indications of spinal pathology	Pathology or disease “such as” / “e.g”	22 (13)
		Specific cause “such as” / “e.g”	22 (13)
		Red flags indicating serious spinal pathology	21 (12)
		Specific cause identified	14 (8)
		Known or suspected serious pathology	10 (6)
		Non-mechanical low back pain	1 (1)
14	Neurological related to the back (symptoms or signs or specific conditions referable to involvement of the spinal cord or nerve roots)	Nerve root compromise	44 (26)
		Neurological signs	34 (20)
		Disc herniation	30 (18)
		Sciatica	17 (10)
		Radicular symptoms	16 (9)
		Signs of nerve root irritation	15 (9)
		Cauda equina syndrome	14 (8)
		Radiation below knee	13 (8)
		Radiculopathy	10 (6)
		Progressive neurological signs	4 (2)
		Radicular pain	4 (2)
		Widespread neurological signs	3 (2)
		Leg symptoms	3 (2)
		Spondylolisthesis with radiculopathy	1 (1)
		Cord compression	1 (1)
		Paralysis	1 (1)
15	Previous or scheduled surgery		84 (50)
16	Pregnancy-related	Pregnancy	81 (48)
		Pre-eclampsia	1 (1)
17	Medico-legal issues		39 (23)
18	Comorbidities	Comorbidities	2 (1)
		Urine or faecal incontinence	1 (1)
19	Exclusion for feasibility	Not understanding language	28 (17)
		Other current treatment	18 (11)
		Previous specific treatment	17 (10)
		Medication	13 (8)
		Contraindications for intervention	8 (5)
		Cardiac pacemaker	6 (4)
		Current alcohol abuse	3 (2)
		Unable to commit to home-exercises	3 (2)
		Fever on the day of examination	3 (2)
		History of psychosis	2 (1)
		History of alcohol abuse	2 (1)
		Presence of yellow flags	1 (1)
20	Miscellaneous	Obesity	
		Body Mass Index ≥30	4 (2)
		Body Mass Index ≥40	3 (2)
		Obesity (not specified)	2 (1)
		Body Mass Index ≥35	1 (1)
		Body Mass Index ≥28	1 (1)
		Menstruation	1 (1)
		Unable to sit on a stationary bike	1 (1)
		Bad balance between trunk flexors and extensors	1 (1)
		Inability to walk at least 100 m without interruption	1 (1)
		Neurologic impairment	1 (1)
		Behaviour precluding participation in group therapy	1 (1)
		Evidence of distress	1 (1)
		Patients unable to accurately locate the area of pain	1 (1)
		Patient unable to walk without a walking aid	1 (1)
		Suspected non-compliance	1 (1)
21	Patient-reported outcome measures with score thresholds for exclusion	Visual Analogue Scale (0 to 100)^c^	
		≥ 60 mm	2 (1)
		≥ 80 mm	2 (1)
		Beck Depression Inventory (0 to 63)	
		≥ 20 points	1 (1)
		≥ 29 points	1 (1)
		Bothersomeness scale (0 to 10)	
		≤ 3 points	1 (1)
		Numeric Rating Scale (0 to 10)	
		≥ 80 points	1 (1)

^a^Bechterew is an eponymous term for ankylosing spondylitis, and was reported in two trials; ^b^Two trials used a banned eponymous term to refer to reactive arthritis. ^c^Visual Analogue Scale transformed to mm

### Inclusion criteria

#### Demographics

While most studies reported the age, gender and symptom duration of their study population, these demographics were variably included as inclusion criteria. Age was reported as selection criterion in 77% of the trials. The lower age entry threshold was specified in 74% of trials and spanned from 15 to 45 years. In contrast, an upper age entry threshold was specified in 64% of trials and spanned from 28 to 80 years. Gender was reported as an inclusion criterion in 13 (8%) trials, either as males (*n* = 3), females (*n* = 2) or both (*n* = 8).

**Fig. 2:**
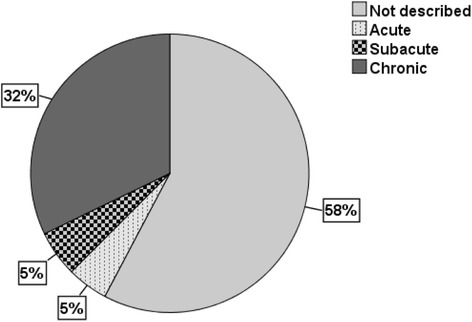
Pie chart of terms used for duration

While most trial reports (*n* = 124, 74%) defined duration of back pain as an inclusion criterion, this ranged from one day to 12 months. Figure 2 shows the proportion of trials that used the terms acute (*n* = 8), subacute (*n* = 9) and chronic (*n* = 54), or did not report any term (*n* = 97). The term ‘chronic’ was used without specifying duration in 8 trials (5%), while pain of at least 3 months (*n* = 27, 16%) was the most commonly specified duration followed by pain of at least 6 months (*n* = 9, 5%).

#### Anatomical area (Table 2, theme one)

Most trial reports (*n* = 143, 85%) did not designate a specific anatomical area as an inclusion criterion. For the 21 (12%) that did, the most common specification was ‘between the costal margin and above gluteal folds’ (*n* = 8, 5%), followed by ‘below scapulae and above gluteal folds’ (*n* = 3, 2%).

#### Symptoms and signs (Table 2, theme two)

While some trial reports were specific when describing pain symptoms (e.g. ‘pain at rest’) used as inclusion criteria, others defined these more generally (e.g. ‘back pain’). Most of the reports described symptoms for inclusion criteria as ‘low back pain’ (*n* = 150, 90%), whereas some used ‘back pain’ even though low back pain was mentioned elsewhere in the paper (*n* = 10, 6%). Further descriptors, such as ‘with or without leg pain’ (*n* = 27, 16%), and ‘pain exacerbated by movement’ (*n* = 5, 3%), were used. Forty-three reports (26%) described more than one symptom, whilst three (2%) used three or more symptoms as inclusion criteria.

### Exclusion criteria

#### Conditions (Table 3, themes one to 12)

Themes one to 12 (i.e. disorders and/or pathologies used as exclusion criteria) and their associated sub-themes could be collectively described as ‘conditions’. Specific conditions were the most commonly described exclusion criteria. Most trials (*n* = 145, 86%) reported at least one condition as an exclusion criterion. The most frequently reported back-related conditions were ‘spondylolisthesis’ (*n* = 35, 21%) and ‘spinal stenosis’ (*n* = 31, 18%).

Specified, unspecified or implied systemic or rheumatologic or immunologic conditions were most commonly referred to using terms such as ‘inflammatory disease’ (*n* = 37, 22%), ‘osteoporosis’ (*n* = 27, 16%), and ‘rheumatologic disease’ (*n* = 13, 8%). LBP due to trauma was described as a condition for exclusion in 63 (37%) trials, most commonly fracture, dislocation and trauma, and major trauma. Psychosocial conditions were exclusion criteria in 58 (34%) trials, mostly described in umbrella terms, such as ‘psychiatric disorder’ (*n* = 35, 20%). Of the excluded conditions related to malignancy, most trials described ‘cancer’, while some reported ‘previous cancer’. Only one trial included ‘significant unexpected weight loss’, as an exclusion criterion.

#### Symptoms, signs and other exclusion criteria (Table 3, themes 13 to 19)

NsLBP was primarily distinguished by exclusion criteria stating that the back pain was not attributable to a ‘specific cause’ (*n* = 22, 13%), or known ‘pathology or disease’ (*n* = 22, 13%), often being described vaguely. The latter were mostly termed in umbrella or over-arching terms, for example ‘red flags indicating serious spinal pathology’ (*n* = 21, 12%) or ‘known or suspected serious pathology’ (*n* = 10, 6%). Several reports (*n* = 44, 26%) listed examples of ‘pathology or disease’ and ‘specific cause’ and while most of these indicated the same conditions, the terms used varied (e.g. tumours, neoplasm and malignancy).

Symptoms and signs included as exclusion criteria were mostly neurological or inferred neurological conditions. The most prevalent exclusion criteria were ‘nerve root compromise’ (*n* = 44, 26%), ‘neurological signs’ (*n* = 34, 20%) and ‘disc herniation’ (*n* = 30, 17%). The same neurological condition was variably described as ‘radicular pain’, ‘radicular symptoms’, ‘radiculopathy’ and ‘sciatica’. Some trial reports (*n* = 32, 19%) described assessment methods to exclude people with neurological signs and symptoms. These were based on radiographic evidence (*n* = 16, 9%) and clinical examination (*n* = 16, 9%); for example, ‘positive Straight Leg Raising, and diminished or decreased motor, sensory, and reflex function’.

Some specific exclusion criteria related to safeguarding the integrity and/or feasibility of the trial. These included exclusion of potential participants who were scheduled for surgery or who had previous surgery related to the back (*n* = 84, 50%), pregnancy (*n* = 81, 48%), medicolegal issues (*n* = 39, 23%), ‘not understanding the language’ (*n* = 28, 16%), ‘other current treatment’ (*n* = 18, 11%) or participant’s prior experience with a given treatment (*n* = 17, 10%).

#### ‘Miscellaneous’ exclusion criteria (Table 3, theme 20)

A ‘miscellaneous’ theme included exclusion criteria that were vague, insofar as the meaning or relevance to nsLBP was unclear, rarely reported, or were less easy to categorise. Obesity was reported as an exclusion criterion in 10 trials (6%) with a Body Mass Index of 30 or more (*n* = 4, 3%), or 40 or more (*n* = 3, 2%) most frequently reported.

Examples of further miscellaneous subthemes include: ‘articular impairment’, ‘menstruation’, ‘being unable to sit on a stationary bike’, ‘bad balance between trunk flexors and extensors’, ‘neurologic impairment’, and individuals with ‘evidence of distress’.

### Patient-reported outcome measure score thresholds (inclusion theme 3 and exclusion theme 21)

Thirty-three trials (20%) included a patient-reported outcome measure score threshold as a selection criterion. The Visual Analogue Scale (VAS) for pain intensity was most utilised as both an inclusion (*n* = 14, 8%) and exclusion criterion (*n* = 4, 2%); the range of VAS score for inclusion was 10 to 65 mm (mean 34 mm) (Table 2), whereas the range of VAS score for exclusion was 60 to 80 mm (mean 70 mm) (Table 3).

## Discussion

Results of this study show that the reported eligibility criteria of people with nsLBP across RCTs is diverse. Trial reports provided relatively fewer details for inclusion criteria than they did for exclusion criteria.

Explicitly reported selection criteria were diverse and only 46% explicitly reported the exclusion of malignancy. However, exclusion of malignancy in the remaining trials may have been assumed to be implied by the definition of nsLBP. Fewer than half of the trials reported neurological compromise as exclusion criteria, whereas only one quarter of trials reported the inclusion of people ‘with or without referred pain’. While most trials reported specific pathologies as exclusion criteria, many of the terms used to describe these were ambiguous or vague, making it difficult to ascertain how these were operationalised. Psychological conditions were reported as exclusion criteria in one-third of trials, which is a surprising finding when the literature proposes that psychological disorders may be a predictor of chronicity in LBP as well as comorbid with pain [31–33]. Use of umbrella or over-arching terms, without specific descriptions of what these were, was frequently seen across all categories of symptoms, signs, and conditions. For example, identification of ‘red flags’ was used as an exclusion criterion in several trials, despite the vagueness of the term (i.e. ‘red flags’ can mean different things) and the weak supporting evidence for red flags determining the presence of a specific cause [34].

Our findings demonstrate the heterogeneity of the selection criteria of RCTs purporting to be studying similar populations. The application of these criteria is typically poorly described, creating difficulty for making judgements on the comparability of study populations. Explicit reporting of clearly defined inclusion and exclusion criteria, using consistent terminology, would increase our confidence in the clinical homogeneity of nsLBP trial populations, increase the validity of meta-analyses, and improve our ability to interpret and compare the results of individual RCTs and systematic reviews.

RCTs that include or exclude people without clear diagnostic criteria or procedure, could lead to including individuals with different prognoses. Without clear, unambiguous descriptions, the utility of reporting exclusion criteria is limited. For example, intervertebral disc herniation is evident in many asymptomatic individuals, and it may often be the case that participants with asymptomatic herniations are admitted into a trial [35, 36].

Of the subthemes of selection criteria that we judged as particularly relevant to back pain, there was inconsistent application between RCTs. For example, spondylolisthesis and spinal stenosis were reported as exclusion criteria in only 20% of trials, but may have been included under the often-reported general indications of spinal pathology such as ‘known or suspected pathology’ or ‘specific cause identified’. Psychosocial conditions were often reported; however, yellow flags were only reported within the selection criteria of one trial [37].

Selection criteria depend on the intervention being tested; there may be good reason for disproportionate focus on biological, psychological or sociological factors. Notwithstanding, over recent decades psychosocial aspects of low back pain have gained much attention [38]. Studies have highlighted the importance of psychosocial factors in the transition from acute to chronic pain; however, we note that few trials used questionnaires designed to assess psychological aspects of pain in relation to the selection of suitable participants for nsLBP trials [38–41].

### Comparisons with existing research

Research on criteria for participating in trials has investigated case definitions and duration of LBP, and specific inclusion and exclusion criteria (e.g. age, though not specifically for nsLBP). These studies show ambiguous presentation of case definitions, duration, and a variation in reported criteria and diagnostic criteria specifically for exclusion of neurological conditions [18, 25, 42]. These results are consistent with our findings. Similar ambiguities have also been described by systematic reviews of trials of interventions for treatment of conditions of the shoulder [43, 44] and neck [45].

Our study empirically corroborates the consensus view of the NIH consortium that clinical studies use variable inclusion/exclusion criteria, and supports the NIH Task Force’s call to develop and draft research standards for chronic low back pain (cLBP) [24]. We hope that this work will be useful in starting and informing discussion surrounding consensus on appropriate entry criteria and what constitutes sufficient detail to adequately describe cLBP study populations.

### Strengths and limitations

This review utilised a systematic multi-reviewer approach and methods developed a priori to review and categorise the selection criteria in nsLBP RCTs. The review has several limitations, which must be acknowledged. The trials investigated were published between 2006 and 2012; thus, more recently published reports are not represented. Prior to 2006 there were changes in the quality of trial reporting following the introduction of CONSORT [46]. Our view is that there is no reason to expect that any large recent change in entry criteria would materially change our findings. The Task Force Report on Research Standards for Chronic Low-Back Pain was published in 2014 and emphasised the variation in inclusion and exclusion criteria, which may have influenced nsLBP trial investigators to become increasingly aware of describing criteria more homogenously [24].

To identify the selection criteria of included trials, we only searched the methods sections. Therefore, it is possible that additional information about selection criteria could have been reported elsewhere in the paper. We also only included English language papers and it is possible (although unlikely) that our findings may not be generalisable to non-English-language reports. We used an iterative method to describe the selection criteria of included trials and merged some categories together for ease of presentation. While other more complex categorisations could have been used, we don’t think is likely to have altered the key findings.

### Recommendations for future nsLBP trials and future research

Following our experience of the challenges of categorising and making a taxonomy of entry criteria domains, we provide in Table 4, for commonly reported domains, examples of the level of detail we suggest is required for the parameters of entry criteria to be sufficiently clear. We offer these suggestions in the spirit of starting and informing discussion surrounding the development of consensus on both clinically relevant domains of entry criteria for LBP trials, and the level of detail required for clarity in the description of how the criterion was operationalised. This approach has worked well previously, where consensus processes have begun with some initial suggestions that are then assessed, scrutinised, and then refined [47, 48]. We confined ourselves here to making suggestions for only commonly reported domains; although empirical research on what has happened in the past may or may not be the most desirable for informing consensus on what should be done in the future. While consistency with what has gone before has value insofar as it facilitates between-trial comparisons, the questions surrounding domain choice and the level of detail that should be provided are principally clinical and in our view, would benefit from separate independent consideration within a Delphi process. [49].

**Table 4:** Suggested examples for the clear reporting of entry criteria, by commonly reported domains

Criterion/a	Examples
Anatomical region	Pain between bottom of ribs and buttock creases
If pain below buttock creases is excluded; state any explicit criteria for exclusions	Pain radiating below knee or objective neurological signs in leg
Age	Adults – with restrictions by age only if good scientific/clinical reason
Duration	Back pain problem that has persisted at least 3 months and has resulted in pain on at least half the days in the past 6 months
Serious causes of LBP excluded	Malignancy, vertebral fracture, infections
Rheumatologic conditions excluded	Ankylosing spondylitis or related conditions
Systemic conditions excluded	Cardiovascular, respiratory, neurological, gastrointestinal, urogenital or related conditions
Baseline severity for studies of treatment (*i.e* not prevention)	NRS/VAS ≥ 4/40, or ≥ 15% of score range of a disability measure (*e.g* 4/24 for RMDQ)
Other patient-characteristics excluded	Pregnancy, medico-legal issues, not understanding language, previous or scheduled surgery, psychiatric disorders
Study specific criteria	Workers or men or with/without depression

Note: The table shows, for commonly reported domains, a series of examples of the level of detail we suggest is required for the parameters of entry criteria to be sufficiently clear

Our paper provides a useful basis for making comparisons with evaluations of nsLBP trials published after 2012 and after the 2014 publication of the NIH report [24]. It will also be important to determine whether there is any improvement in the reporting of selection criteria in future nsLBP trials based upon this paper and our suggested template.

## Conclusion

Inclusion and exclusion criteria in RCTs of nsLBP are diverse, and terms and descriptions used are inconsistent and often described ambiguously using over-arching terms. The use of more consistent selection criteria and unambiguous reporting of these should improve population homogeneity between trials, facilitating comparisons and meta-analyses. We offer a template of criteria as a starting point that may be adapted, if required, depending on the intervention under investigation.

## Additional files

Additional file 1: Typical search strategy. Database search strategy. (DOCX 14 kb)

Additional file 2: Excluded and included trials. References to excluded and included trials. (DOCX 45 kb)

Additional file 3: **Table S1.** Included study characteristics. (PDF 490 kb)

Additional file 4: **Table S2.** Excluded study characteristics. (PDF 300 kb)

## Abbreviations

CI: Confidence Interval
LBP: Low Back Pain
RCT: Randomised Controlled Trial
YLD: Years Lived with Disability
