# Supplementary material for: Inclusion and exclusion criteria used in non-specific low back pain trials: a review of randomised controlled trials published between 2006 and 2012
Source: BMC Musculoskelet Disord. 2018 Apr 12;19:113. doi: 10.1186/s12891-018-2034-6 (PMC5898037; doi:10.1186/s12891-018-2034-6)
Supplement: Supplementary file 1 — Typical search strategy. Database search strategy. (DOCX 14 kb) [file 12891_2018_2034_MOESM1_ESM.docx]

Typical search strategy PubMed

#1 (("2007/1/1"[Date - MeSH] : "3000"[Date - MeSH])) AND low back pain

#2 (("2007/1/1"[Date - MeSH] : "3000"[Date - MeSH])) AND back pain

#3 low back pain OR lumbago OR backache

#4 (#1 OR #2 OR #3) Filters: Clinical Trial; Publication date from 2007/01/01 to 2012/01/01
