# Supplementary material for: Inclusion and exclusion criteria used in non-specific low back pain trials: a review of randomised controlled trials published between 2006 and 2012
Source: BMC Musculoskelet Disord. 2018 Apr 12;19:113. doi: 10.1186/s12891-018-2034-6 (PMC5898037; doi:10.1186/s12891-018-2034-6)
Supplement: Supplementary file 2 — Excluded and included trials. References to excluded and included trials. (DOCX 45 kb) [file 12891_2018_2034_MOESM2_ESM.docx]

1. Anon: Erratum: Efficacy and safety of tapentadol extended release for the management of chronic low back pain: results of a prospective, randomized, double-blind, placebo- and active-controlled phase iii study. Expert Opin. Pharmacother. 11(16), 2773 (2010)
2. Ansari, N.N., Ebadi, S., Talebian, S., Naghdi, S., Mazaheri, H., Olyaei, G., Jalaie, S.: A randomized, single blind placebo controlled clinical trial on the effect of continuous ultrasound on low back pain. Electromyogr Clin Neurophysiol 46(6), 329-336 (2006)
3. Barker, K.L., Elliott, C.J., Sackley, C.M., Fairbank, J.C.: Treatment of chronic back pain by sensory discrimination training. a phase i rct of a novel device (fairmed) vs. tens. BMC Musculoskelet Disord 9, 97 (2008)
4. Basler, H.D., Bertalanffy, H., Quint, S., Wilke, A., Wolf, U.: Ttm-based counselling in physiotherapy does not contribute to an increase of adherence to activity recommendations in older adults with chronic low back pain-a randomised controlled trial. Eur J Pain 11(1), 31-7 (2007)
5. Belavy, D.L., Armbrecht, G., Gast, U., Richardson, C.A., Hides, J.A., Felsenberg, D.: Countermeasures against lumbar spine deconditioning in prolonged bed rest: resistive exercise with and without whole body vibration. J Appl Physiol 109(6), 1801-11 (2010)
6. Bergholdt, K., Fabricius, R.N., Bendix, T.: Better backs by better beds? Spine (Phila Pa 1976) 33(7), 703-8 (2008)
7. Bruehl, S., Burns, J.W., Chung, O.Y., Quartana, P.: Anger management style and emotional reactivity to noxious stimuli among chronic pain patients and healthy controls: the role of endogenous opioids. Health Psychol 27(2), 204-14 (2008)
8. Bruehl, S., Chung, O.Y., Burns, J.W., Diedrich, L.: Trait anger expressiveness and pain-induced beta-endorphin release: support for the opioid dysfunction hypothesis. Pain 130(3), 208-15 (2007)
9. Buttagat, V., Eungpinichpong, W., Chatchawan, U., Kharmwan, S.: The immediate effects of traditional Thai massage on heart rate variability and stress-related parameters in patients with back pain associated with myofascial trigger points (2011)
10. Buynak, R., Etropolski, M., Lange, B., Shapiro, D.Y., Okamoto, A., Steup, A., Van Hove, I.: Dose stability of tapentadol er for the relief of chronic low back pain: Results of a randomized, active- and placebo-controlled study. Arthritis and Rheumatism 60, 1494 (2009)
11. Cabitza, P., Randelli, P.: Effcacy and safety of eperisone in patients with low back pain: a double blind randomized study. Eur Rev Med Pharmacol Sci 12(4), 229-35 (2008)
12. Casserley-Feeney S, H.-O.D.A.: The ACCESS trial - randomised controlled trial of public hospital-based versus private clinic-based physiotherapy for low back pain: clinical outcomes (2007)
13. Cevik, R., Bilici, A., Gur, A., Sarac, A.J., Yildiz, H., Nas, K., Ceviz, A., Bukte, Y.: Effect of new traction technique of prone position on distraction of lumbar vertebrae and its relation with different application of heating therapy in low back pain (2007)
14. Christiansen, S., Oettingen, G., Dahme, B., Klinger, R.: A short goal-pursuit intervention to improve physical capacity: A randomized clinical trial in chronic back pain patients. Pain 149(3), 444-452 (2010)
15. Clark, D., Chu, L.: Tolerance and opioid-induced hyperalgesia in clinical populations. European Journal of Pain Supplements 4(1), 29 (2010)
16. Cleland, J., Fritz, J., Kulig, K., Davenport, T.E., Eberhart, S., Magel, J.S., Childs, J.D.: Comparison of the effectiveness of 3 manual physical therapy techniques in a subgroup of patients with low back pain who satisfy a clinical prediction rule: a randomized clinical trial (2009)
17. Codding, C., Levinsky, D., Hale, M.E., Thomas, J.W., Lockhart, E., Best, A., Jain, R.: Efficacy and safety evaluation of 12 weeks extended-release hydrocodone/acetaminophen treatment in patients with chronic low back pain (clbp) by prior opioid use. Pain Medicine 10(1), 260 (2009)
18. Cohen, S.P., Stojanovic, M.P., Crooks, M., Kim, P., Schmidt, R.K., Shields, C.H., Croll, S., Hurley, R.W.: Lumbar zygapophysial (facet) joint radiofrequency denervation success as a function of pain relief during diagnostic medial branch blocks: a multicenter analysis. Spine Journal 8(3), 498-504 (2008)
19. Cox, J.M.: A randomized controlled trial comparing 2 types of spinal manipulation and minimal conservative medical care for adults 55 years and older with subacute or chronic low back pain. J Manipulative Physiol Ther 32(7), 601 (2009)
20. Curnow, D., Cobbin, D., Wyndham, J., Boris Choy, S.T.: Altered motor control, posture and the pilates method of exercise prescription. J Bodyw Mov Ther 13(1), 104-111 (2009)
21. Dagenais, S., Yelland, M.J., Del Mar, C., Schoene, M.L.: Prolotherapy injections for chronic low-back pain. Cochrane Database Syst Rev (2), 004059 (2007)
22. Day, I.J., Kent, C.F., Burnham, R.S.: Can topical anesthetic reduce the pain associated with diagnostic blocks of the lumbosacral spine? Pain Med 9(6), 675-9 (2008)
23. Demoulin, C., Maquet, D., Tomasella, M., Croisier, J., Crielaard, J., Vanderthommen, M.: Benefits of a physical training program after back school for chronic low back pain patients 14(2), 21-31 (2006)
24. Deshpande, A., Furlan, A., Mailis-Gagnon, A., Atlas, S., Turk, D.: Opioids for chronic low-back pain. Cochrane Database Syst Rev (3), 004959 (2007)
25. Desmoulin, G.T., Yasin, N.I., Chen, D.W.: Initial results using khan kinetic treatment(trademark) as a low back pain treatment option. Journal of Musculoskeletal Pain 15(3), 91-102 (2007)
26. Engers, A., Jellema, P., Wensing, M., van der Windt, D.A., Grol, R., van Tulder, M.W.: Individual patient education for low back pain. Cochrane Database Syst Rev (1), 004057 (2008)
27. Etropolski, M., Rauschkolb-Loffler, C., Shapiro, D., Okamoto, A., Lange, C.: A randomized, double-blind, placebo- and active-controlled phase iii study of tapentadol er for chronic low back pain: Analysis of efficacy endpoint sensitivity. Journal of Pain 10(4), 51 (2009)
28. Etropolski, M.S., Okamoto, A., Shapiro, D.Y., Rauschkolb, C.: Dose conversion between tapentadol immediate and extended release for low back pain. Pain Physician 13(1), 61-70 (2010)
29. Evans, D.D., Carter, M., Panico, R., Kimble, L., Morlock, J.T., Spears, M.J.: Characteristics and predictors of short-term outcomes in individuals self-selecting yoga or physical therapy for treatment of chronic low back pain. PM and R 2(11), 1006-1015 (2010)
30. Gatti, R., Faccendini, S., Tettamanti, A., Barbero, M., Balestri, A., Calori, G.: Efficacy of trunk balance exercises for individuals with chronic low back pain: a randomized clinical trial. J Orthop Sports Phys Ther 41(8), 542-52 (2011)
31. George, S.Z., Childs, J.D., Teyhen, D.S., Wu, S.S., Wright, A.C., Dugan, J.L., Robinson, M.E.: Brief psychosocial education, not core stabilization, reduced incidence of low back pain: results from the prevention of low back pain in the military (polm) cluster randomized trial. BMC Med 9, 128 (2011)
32. George, S.Z., Teyhen, D.S., Wu, S.S., Wright, A.C., Dugan, J.L., Yang, G., Robinson, M.E., Childs, J.D.: Psychosocial education improves low back pain beliefs: results from a cluster randomized clinical trial (nct00373009) in a primary prevention setting. Eur Spine J 18(7), 1050-8 (2009)
33. George, S.Z., Wittmer, V.T., Fillingim, R.B., Robinson, M.E.: Comparison of graded exercise and graded exposure clinical outcomes for patients with chronic low back pain. J Orthop Sports Phys Ther 40(11), 694-704 (2010)
34. Gould, E.M., Jensen, M.P., Victor, T.W., Gammaitoni, A.R., White, R.E., Galer, B.S.: The pain quality response profile of oxymorphone extended release in the treatment of low back pain. Clin J Pain 25(2), 116-22 (2009)
35. Guthrie, R.J., Grindstaff, T.L., Croy, T., Ingersoll, C.D., Saliba, S.A.: The effect of traditional bridging or suspension exercise bridging on lateral abdominal thickness in individuals with low back pain. J Sport Rehabil (2011)
36. Hale, M., Khan, A., Kutch, M., Li, S.: Once-daily oros hydromorphone er compared with placebo in opioid-tolerant patients with chronic low back pain. Curr Med Res Opin 26(6), 1505-18 (2010)
37. Hale, M.E., Ahdieh, H., Ma, T., Rauck, R.: Efficacy and safety of opana er (oxymorphone extended release) for relief of moderate to severe chronic low back pain in opioid-experienced patients: a 12-week, randomized, double-blind, placebo-controlled study. J Pain 8(2), 175-84 (2007)
38. Hancock: letter (2010)
39. Hasegawa, T.M., Baptista, A.S., De Souza, M.C., Yoshizumi, A.M., Natour, J.: Acupuncture for acute non-specifc low back pain: A randomized, controlled, placebo trial. Arthritis and Rheumatism 60, 1497 (2009)
40. Helmhout, P.H., Harts, C.C., Viechtbauer, W., Staal, J.B., de Bie, R.A.: Isolated lumbar extensor strengthening versus regular physical therapy in an army working population with nonacute low back pain: a randomized controlled trial. Arch Phys Med Rehabil 89(9), 1675-85 (2008)
41. Helmhout, P.H., Staal, J.B., Heymans, M.W., Harts, C.C., Hendriks, E.J., de Bie, R.A.: Prognostic factors for perceived recovery or functional improvement in non-specific low back pain: secondary analyses of three randomized clinical trials. Eur Spine J 19(4), 650-9 (2010)
42. Henchoz, Y., Pinget, C., Wasserfallen, J.B., Paillex, R., de Goumoens, P., Norberg, M., Kai-Lik So, A.: Cost-utility analysis of a three-month exercise programme vs usual care following multidisciplinary rehabilitation for chronic low back pain. J Rehabil Med 42(9), 846{52 (2010)
43. Hides, J.A., Stanton, W.R., Mendis, M.D., Gildea, J., Sexton, M.J.: Effect of motor control training on muscle size and football games missed from injury. Med Sci Sports Exerc (2011)
44. Hlobil, H., Uegaki, K., Staal, J.B., de Bruyne, M.C., Smid, T., van Mechelen, W.: Substantial sick-leave costs savings due to a graded activity intervention for workers with non-specific sub-acute low back pain. Eur Spine J 16(7), 919-24 (2007)
45. Hollinghurst, S., Sharp, D., Ballard, K., Barnett, J., Beattie, A., Evans, M., Lewith, G., Middleton, K., Oxford, F., Webley, F., Little, P.: Randomised controlled trial of alexander technique lessons, exercise, and massage (ateam) for chronic and recurrent back pain: economic evaluation. BMJ 337, 2656 (2008)
46. Hurley, D.A., O'Donoghue, G., Tully, M.A., Moffett, J.K., van Mechelen, W., Daly, L., Boreham, C.A., McDonough, S.M.: A walking programme and a supervised exercise class versus usual physiotherapy for chronic low back pain: a single-blinded randomised controlled trial. (the supervised walking in comparison to fitness training for back pain (swift) trial). BMC Musculoskelet Disord 10, 79 (2009)
47. Hush, J.: Tens of unknown value in the treatment of chronic low back pain. Aust J Physiother 52(1), 64 (2006)
48. Ijzelenberg, H., Meerding, W.J., Burdorf, A.: Effectiveness of a back pain prevention program: A cluster randomized controlled trial in an occupational setting. Spine 32(7), 711-719 (2007)
49. Ikegami, S., Kamimura, M., Uchiyama, S., Nakagawa, H., Hashidate, H., Takahara, K., Takahashi, J., Kato,H.: Anti-nociceptive effects of elcatonin injection for postmenopausal women with back pain: A randomized controlled trial. Osteoporosis International 21, 197-198 (2010)
50. Inoue, M., Hojo, T., Nakajima, M., Kitakoji, H., Itoi, M.: Comparison of the effectiveness of acupuncture treatment and local anaesthetic injection for low back pain: a randomised controlled clinical trial. Acupunct Med 27(4), 174-7 (2009)
51. Jans, M.P., Korte d, E.M., Heinrich, J., Hildebrandt, V.H.: Intermittent follow-up treatment with Cesar exercise therapy in patients with subacute or chronic aspecific low back pain: results of a randomized, controlled trial with a 1.5-year follow-up (2006)
52. Kasis, A.G., Marshman, L.A.G., Krishna, M., Bhatia, C.K.: Significantly improved outcomes with a less invasive posterior lumbar interbody fusion incorporating total facetectomy (2009)
53. Katz, N., Borenstein, D., Birbara, C., Bramson, C., Nemeth, M., Smith, M., Brown, M.: Tanezumab, an anti-nerve growth factor (ngf) antibody, for the treatment of chronic low back pain (clbp) - a randomized, controlled, double-blind, phase 2 trial. Journal of Pain 10(4), 42 (2009)
54. Katz, N., Rauck, R., Ahdieh, H., Ma, T., Van Der Hoop, R.G., Kerwin, R., Podolsky, G.: A 12-week, randomized, placebo-controlled trial assessing the safety and efficacy of oxymorphone extended release for opioid-naive patients with chronic low back pain. Current Medical Research and Opinion 23(1), 117-128 (2007)
55. Kavanagh, S., Lange, B., Ashworth, J., Etropolski, M.S., McNeill, M., Rauschkolb, C.: Tapentadol extended release (er) for chronic low back pain: Results of euroqol-5 dimension (eq-5d) and short form-36 (sf-36) health status questionnaires. Value in Health 12(7), 376 (2009)
56. Kettenmann, B., Wille, C., Lurie-Luke, E., Walter, D., Kobal, G.: Impact of continuous low level heatwrap therapy in acute low back pain patients: subjective and objective measurements. Clin J Pain 23(8), 663-8 (2007)
57. Kool, J., Bachmann, S., Oesch, P., Knuesel, O., Ambergen, T., de Bie, R., van den Brandt, P.: Function-centered rehabilitation increases work days in patients with nonacute nonspecific low back pain: 1-year results from a randomized controlled trial. Arch Phys Med Rehabil 88(9), 1089-94 (2007)
58. Kovacs, F., Abraira, V., Santos, S., Diaz, E., Gestoso, M., Muriel, A., Gil del Real, M.T., Mufraggi, N., Noguera, J., Zamora, J.: A comparison of two short education programs for improving low back pain-related disability in the elderly: a cluster randomized controlled trial. Spine (Phila Pa 1976) 32(10), 1053-9 (2007)
59. Kullich, W., Schwann, H., Machreich, K., Ausserwinkler, M.: Additional outcome improvement in the rehabilitation of chronic low back pain after nuclear resonance therapy. Rheumatologia 20(1), 7-12 (2006)
60. Kullich, W., Schwann, H., Walcher, J., Machreich, K.: The effect of MBST-Nuclear Resonance Therapy with a complex 3-dimensional electromagnetic nuclear resonance field on patients with low back pain 23 (2006)
61. Lamb, S.E., Lall, R., Hansen, Z., Castelnuovo, E., Withers, E.J., Nichols, V., Griffiths, F., Potter, R., Szczepura, A., Underwood, M.: A multicentred randomised controlled trial of a primary care-based cognitive behavioural programme for low back pain. the back skills training (best) trial. Health Technol Assess 14(41), 1-253 (2010)
62. Lee, J.W., Shin, H.I., Park, S.Y., Lee, G.Y., Kang, H.S.: Therapeutic trial of fluoroscopic interlaminar epidural steroid injection for axial low back pain: effectiveness and outcome predictors. AJNR Am J Neuroradiol 31(10), 1817-23 (2010)
63. Lee, T.J.: Pharmacologic treatment for low back pain: one component of pain care. Phys Med Rehabil Clin N Am 21(4), 793{800 (2010)
64. Leichtfried, V., Kantner-Rumplmair, W., Raggautz, M., Bartenbach, C., Aigner, M., Winkler, D., Jonas, L., Gehmacher, D., Schobersberger, W.: Can bright light therapy ameliorate symptoms associated with low back pain (LBP)? A randomized controlled trial. Journal of Psychosomatic Research 68(6), 642 (2010)
65. Lewis, C., Khan, A., Souvlis, T., Sterling, M.: A randomised controlled study examining the short-term effects of strain-counterstrain treatment on quantitative sensory measures at digitally tender points in the low back. Man Ther 15(6), 536-41 (2010)
66. Li, C., Ni, J., Wang, Z., Li, M., Gasparic, M., Terhaag, B., Uberall, M.A.: Analgesic efficacy and tolerability of flupirtine vs. tramadol in patients with subacute low back pain: a double-blind multicentre trial. Curr Med Res Opin 24(12), 3523-30 (2008)
67. Dianne Liddle, S. et al. Advice for the management of low back pain: A systematic review of randomised controlled trials Manual Therapy , Volume 12 , Issue 4 , 310 - 327
68. Long, A., May, S., Fung, T.: The comparative prognostic value of directional preference and centralization: A useful tool for front-line clinicians? Journal of Manual and Manipulative Therapy 16(4), 248-254 (2008)
69. Macfarlane, G.J.: Changing patient perceptions of their illness: Can they contribute to an improved outcome for episodes of musculoskeletal pain? Pain 136(1-2), 1-2 (2008)
70. Magnusson, M.L., Chow, D.H., Diamandopoulos, Z., Pope, M.H.: Motor control learning in chronic low back pain. Spine (Phila Pa 1976) 33(16), 532-8 (2008)
71. Mandara, A., Fusaro, A., Musicco, M., Bado, F.: A randomised controlled trial on the effectiveness of osteopathic manipulative treatment of chronic low back pain. Int J Ost Med 11(4), 156 (2008)
72. Mattila, R., Malmivaara, A., Kastarinen, M., Kivela, S.L., Nissinen, A.: The effects of lifestyle intervention for hypertension on low back pain: a randomized controlled trial. Spine (Phila Pa 1976) 32(26), 2943-7 (2007)
73. Mehling, W.E.: Breath therapy for chronic low back pain. J Bodyw Mov Ther 10(2), 96-8 (2006)
74. Mehta, S., Chopra, A., Goregaonkar, A., Chandanwale, A., Medhi, B., Shah, V., Langade, D., Maroli, S., Gaikwad, S., Pawar, D.: Evaluation of efficacy and safety of eperisone hydrochloride in treatment of acute musculoskeletal spasm associated with low back pain: A randomized, doubleblind, placebo-controlled trial. Pain Practice 9, 123 (2009)
75. Meng, K., Seekatz, B., Roband, H., Worringen, U., Vogel, H., Faller, H.: Intermediate and long-term effects of a standardized back school for inpatient orthopedic rehabilitation on illness knowledge and self-management behaviors: a randomized controlled trial. Clin J Pain 27(3), 248-57 (2011)
76. Mirovsky, Y., Grober, A., Blankstein, A., Stabholz, L.: The effect of ambulatory lumbar traction combined with treadmill on patients with chronic low back pain. Journal of Back and Musculoskeletal Rehabilitation 19(2-3), 73-78 (2006)
77. Miyazaki, S., Hagihara, A., Kanda, R., Mukaino, Y., Nobutomo, K.: Applicability of press needles to a double-blind trial: a randomized, double-blind, placebo-controlled trial. Clin J Pain 25(5), 438-44 (2009)
78. Murtezani, A., Hundozi, H., Orovcanec, N., Sllamniku, S., Osmani, T.: A comparison of high intensity aerobic exercise and passive modalities for the treatment of workers with chronic low back pain: a randomized, controlled trial. Eur J Phys Rehabil Med 47(3), 359-66 (2011)
79. Najm, W.I.: German acupuncture trials (gerac) for chronic low back pain. Medical Acupuncture 20(2), 131-132 (2008)
80. Nath, S., Nath, C.A., Pettersson, K.: Percutaneous lumbar zygapophysial (facet) joint neurotomy using radiofrequency current, in the management of chronic low back pain: a randomized double-blind trial. Spine (Phila Pa 1976) 33(12), 1291-71298 (2008)
81. Nelson-Wong, E., Callaghan, J.P.: Changes in muscle activation patterns and subjective low back pain ratings during prolonged standing in response to an exercise intervention. J Electromyogr Kinesiol. 20(6), 1125-33 (2010)
82. North, R.B., Kidd, D.H., Olin, J., Sieracki, J.M., Boulay, M.: Spinal cord stimulation with interleaved pulses: A randomized, controlled trial. Neuromodulation 10(4), 349-357 (2007)
83. O'Brien, N., Hanlon, M., Meldrum, D.: Randomised, controlled trial comparing physiotherapy and Pilates in the treatment of ordinary low back pain 452 3165 (2006)
84. O'Donnell, J.B., Ekman, E.F., Spalding, W.M., Bhadra, P., McCabe, D., Berger, M.F.: The effectiveness of a weak opioid medication versus a cyclo-oxygenase-2 (cox-2) selective non-steroidal anti-inflammatory drug in treating are-up of chronic low-back pain: results from two randomized, double-blind, 6-week studies. J Int Med Res 37(6), 1789-802 (2009)
85. Padua, R., Bondi, R., Ceccarelli, E., Alviti, F.: Re: A randomized study of back school in women with chronic low back pain. quality of life at three, six, and twelve months follow-up. Spine (Phila Pa 1976) 34(12), 1336 (2009)
86. Pareek, A., Chandurkar, N., Chandanwale, A.S., Ambade, R., Gupta, A., Bartakke, G.: Aceclofenac-tizanidine in the treatment of acute low back pain: a double-blind, double-dummy, randomized, multicentric, comparative study against aceclofenac alone. Eur Spine J 18(12), 1836-42 (2009)
87. Pengel, L.H., Refshauge, K.M., Maher, C.G., Nicholas, M.K., Herbert, R.D., McNair, P.: Physiotherapist-directed exercise, advice, or both for subacute low back pain: a randomized trial. Ann Intern Med 146(11), 787-96 (2007)
88. Peniston, J.H., Gould, E.: Oxymorphone extended release for the treatment of chronic low back pain: a retrospective pooled analysis of enriched-enrollment clinical trial data stratified according to age, sex, and prior opioid use. Clin Ther 31(2), 347-59 (2009)
89. Perrot, S., Krause, D., Crozes, P., Naim, C.: Efficacy and tolerability of paracetamol/tramadol (325 mg/37.5 mg) combination treatment compared with tramadol (50 mg) monotherapy in patients with subacute low back pain: a multicenter, randomized, double-blind, parallel-group, 10-day treatment study. Clin Ther 28(10), 1592-606 (2006)
90. Petersen, T., Larsen, K., Nordsteen, J., Olsen, S., Fournier, G., Jacobsen, S.: The mckenzie method compared with manipulation when used adjunctive to information and advice in low back pain patients presenting with centralization or peripheralization: a randomized controlled trial. Spine (Phila Pa 1976) 36(24), 1999-2010 (2011)
91. Petrofsky, J.S., Batt, J., Brown, J., Stacey, L., Bartelink, T., Le Moine, M., Charbonnet, M., Leyva, S., Lohman, E.B., Aiyar, S., Christensen, A., Weis, D., Weis, M., Jackson, J., Rad-Bayani, E., Prowse, M., Sharma, A., Rendon, A.: Improving the outcomes after back injury by a core muscle strengthening program. J Appl Res 8(1), 62{75 (2008)
92. Podichetty, V.K., Varley, E.S.: Re: Oleske d m, lavender s a, andersson g b, et al. are back supports plus education more effective than education alone in promoting recovery from low back pain? results from a randomized clinical trial. spine 2007;32:2050-7. Spine (Phila Pa 1976) 33(3), 349-50 (2008)
93. Popovic, D.B., Bijelic, G., Miler, V., Dosen, S., Popovic, M.B., Schwirtlich, L.: Lumbar stimulation belt for therapy of low-back pain. Artif Organs 33(1), 54-60 (2009)
94. Portenoy, R.K., Messina, J., Xie, F., Peppin, J.: Fentanyl buccal tablet (fbt) for relief of breakthrough pain in opioid-treated patients with chronic low back pain: a randomized, placebo-controlled study. Curr Med Res Opin 23(1), 223-33 (2007)
95. del Pozo-Cruz B1, Hernández Mocholí MA, Adsuar JC, Parraca JA, Muro I, Gusi N. Effects of whole body vibration therapy on main outcome measures for chronic non-specific low back pain: a single-blind randomized controlled trial. J Rehabil Med. 2011 Jul;43(8):689-94
96. Pushpika Attanayake, A.M., Somarathna, K.I., Vyas, G.H., Dash, S.C.: Clinical evaluation of selected yogic procedures in individuals with low back pain. Ayu 31(2), 245-50 (2010)
97. Quartana, P.J., Burns, J.W., Loffland, K.R.: Attentional strategy moderates effects of pain catastrophizing on symptom-specific physiological responses in chronic low back pain patients. J Behav Med 30(3), 221-31 (2007)
98. Ralph, L., Look, M., Wheeler, W., Sacks, H.: Double-blind, placebo-controlled trial of carisoprodol 250-mg tablets in the treatment of acute lower-back spasm. Curr Med Res Opin 24(2), 551-8 (2008)
99. Ralph, L., Wheeler, B., Sacks, H.: Improvement in functional status with carisoprodol 250-mg tablets in patients with acute lower back spasm: A randomized, double-blind, placebo-controlled trial. Pain Medicine 10(1), 258 (2009)
100. Rauck, R.L., Bookbinder, S.A., Bunker, T.R., Alftine, C.D., Ghalie, R., Negro-Vilar, A., de Jong, E., Gershon, S.: The action study: a randomized, open-label, multicenter trial comparing once-a-day extended-release morphine sulfate capsules (avinza) to twice-a-day controlled-release oxycodone hydrochloride tablets (oxycontin) for the treatment of chronic, moderate to severe low back pain. J Opioid Manag 2(3), 155-66 (2006)
101. Rauck, R.L., Bookbinder, S.A., Bunker, T.R., Alftine, C.D., Ghalie, R., Negro-Vilar, A., de Jong, E., Gershon, S.: A randomized, open-label study of once-a-day avinza (morphine sulfate extended-release capsules) versus twice-a-day oxycontin (oxycodone hydrochloride controlled-release tablets) for chronic low back pain: the extension phase of the action trial. J Opioid Manag 2(6), 325-83313 (2006)
102. Rivero Arias, O., Gray, A., Frost, H., Lamb, S.E., Stewart Brown, S.: Cost-utility analysis of physiotherapy treatment compared with physiotherapy advice in low back pain. Spine. 31(12), 1381-7 (2006)
103. Romano, C.L., Romano, D., Bonora, C., Mineo, G.: Pregabalin, celecoxib, and their combination for treatment of chronic low-back pain. J Orthop Traumatol 10(4), 185-91 (2009)
104. Rusinyol, F.C., Perice, R.V., Boronat, E.R., Bosch, F.F.: Effects of two different doses of eperisone in thetreatment of acute low back pain. Journal of Applied Research 9(1-2), 23-29 (2009)
105. Ruth, M., Weber, M., Zenz, M.: Laser acupuncture for chronic back pain. a double-blind clinical study. Schmerz 24(5), 485-493 (2010)
106. Schimmel, J.J., de Kleuver, M., Horsting, P.P., Spruit, M., Jacobs, W.C., van Limbeek, J.: No effect of traction in patients with low back pain: a single centre, single blind, randomized controlled trial ofintervertebral differential dynamics therapy. Eur Spine J 18(12), 1843-50 (2009)
107. Schmidt-Wilcke, T.: Affective components and intensity of pain correlate with structural differences in gray matter in chronic back pain patients. Pain 125(1-2), 89-97 (2006)
108. Schwarz, I., Lawrence, D.J.: Relative responsiveness of 3 different types of clinical outcome measures on chiropractic patients with low back pain. Journal of Manipulative and Physiological Therapeutics 30(1), 77-78 (2007)
109. Serfer, G.T., Wheeler, W.J., Sacks, H.J.: Randomized, double-blind trial of carisoprodol 250 mg compared with placebo and carisoprodol 350 mg for the treatment of low back spasm. Curr Med Res Opin 26(1), 91-9 (2010)
110. Sertpoyraz, F., Eyigor, S., Karapolat, H., Capaci, K., Kirazli, Y.: Comparison of isokinetic exercise versus standard exercise training in patients with chronic low back pain: a randomized controlled study. Clin Rehabil 23(3), 238{47 (2009)
111. Shakoor, M.A., Salek, A.K.M., Islam, M.T., Moyeenuzzaman, M.: Evaluation of the effects of selective rehabilitation on the patients with chronic low back pain. International Journal of Rheumatic Diseases 13, 221 (2010)
112. Sherman, K.J., Cherkin, D.C., Ichikawa, L., Avins, A.L., Barlow, W.E., Khalsa, P.S., Deyo, R.A.: Characteristics of patients with chronic back pain who benefit from acupuncture. BMC Musculoskelet Disord 10, 114 (2009)
113. Sherman, K.J., Cherkin, D.C., Ichikawa, L., Avins, A.L., Delaney, K., Barlow, W.E., Khalsa, P.S., Deyo, R.A.: Treatment expectations and preferences as predictors of outcome of acupuncture for chronic back pain. Spine (Phila Pa 1976) 35(15), 1471-7 (2010)
114. Shimoji, K., Takahashi, N., Nishio, Y., Koyanagi, M., Aida, S.: Pain relief by transcutaneous electric nerve stimulation with bidirectional modulated sine waves in patients with chronic back pain: a randomized, double-blind, sham-controlled study. Neuromodulation 10(1), 42-51 (2007)
115. Shum, G.: Movement coordination of the lumbar spine and hip during a picking up activity in low back pain subjects. Eur Spine J 16(6), 749-58 (2006)
116. Skljarevski, V., Zhang, S., Desaiah, D., Palacios, S., Miazgowski, T., Patrick, K.: Efficacy and safety of duloxetine 60 mg once-daily in patients with chronic low back pain. Journal of Pain 11(4), 38 (2010)
117. Skljarevski, V., Zhang, S., Desaiah, D., Palacios, S., Miazgowski, T., Patrickm, K.: Effect of duloxetine 60 mg once daily versus placebo in patients with chronic low back pain: A 12-week, randomized, double-blind trial. Pain Medicine 11(2), 322 (2010)
118. Slater, M.A., Weickgenant, A.L., Greenberg, M.A., Wahlgren, D.R., Williams, R.A., Carter, C., Patterson, T.L., Grant, I., Garfin, S.R., Webster, J.S., Atkinson, J.H.: Preventing progression to chronicity in first onset, subacute low back pain: an exploratory study. Arch Phys Med Rehabil 90(4), 545-52 (2009)
119. Smeets, R.J.: Do lumbar stabilising exercises reduce pain and disability in patients with recurrent low back pain? Aust J Physiother 55(2), 138 (2009)
120. Smeets, R.J., Vlaeyen, J.W., Hidding, A., Kester, A.D., van der Heijden, G.J., Knottnerus, J.A.: Chronic low back pain: physical training, graded activity with problem solving training, or both? the one-year post-treatment results of a randomized controlled trial. Pain 134(3), 263-76 (2008)
121. Smeets, R.J.E.M., Beelen, S., Goossens, M.E.J.B., Schouten, E.G.W., Knottnerus, J.A., Vlaeyen, J.W.S.: Treatment expectancy and credibility are associated with the outcome of both physical and cognitive-behavioral treatment in chronic low back pain. Clinical Journal of Pain 24(4), 305-315 (2008)
122. Smith, A.L., Kolt, G.S., McConville, J.C.: The effect of the felenkrais method on pain and anxiety in people experiencing chronic low back pain 3136. New Zealand Journal of Physiotherapy 29(1), 6-14 (2007)
123. Sokunbi, O., Watt, P., Moore, A.: Changes in plasma concentration of serotonin in response to spinal stabilisation exercises in chronic low back pain patient. Nig Q J Hosp Med 17(3), 108-11 (2007)
124. Soonawalla, D.F., Joshi, N.: Efficacy of thiocolchicoside in indian patients suffering from low back pain associated with muscle spasm. J Indian Med Assoc 106(5), 331-5 (2008)
125. Steiner, D., Munera, C., Hale, M., Ripa, S., Landau, C.: The efficacy and safety of buprenorphine transdermal system (BTDS) in subjects with moderate to severe low back pain: A double-blind study. Journal of Pain 10(4), 51 (2009)
126. Steiner, D., Munera, C., Hale, M., Ripa, S., Landau, C.: Efficacy and safety of buprenorphine transdermal system (BTDS) for chronic moderate to severe low back pain: a randomized, double-blind study. J Pain 12(11), 1163-73 (2011)
127. Steiner, D.J., Sitar, S., Wen, W., Sawyerr, G., Munera, C., Ripa, S.R., Landau, C.: Efficacy and safety of the seven-day buprenorphine transdermal system in opioid-naive patients with moderate to severe chronic low back pain: an enriched, randomized, double-blind, placebo-controlled study. J Pain Symptom Manage 42(6), 903-17 (2011)
128. Sutlive, T.G., Mabry, L.M., Easterling, E.J., Durbin, J.D., Hanson, S.L., Wainner, R.S., Childs, J.D.: Comparison of short-term response to two spinal manipulation techniques for patients with low back pain in a military beneficiary population. Mil Med 174(7), 750-6 (2009)
129. Tavafian, S.S., Jamshidi, A.R., Montazeri, A.: A randomized study of back school in women with chronic low back pain: quality of life at three, six, and twelve months follow-up. Spine (Phila Pa 1976) 33(15), 1617-21 (2008)
130. Tavafian, S.S., Jamshidi, A.R., Mohammad, K.: Treatment of chronic low back pain: a randomized clinical trial comparing multidisciplinary group-based rehabilitation program and oral drug treatment with oral drug treatment alone. Clin J Pain 27(9), 811-8 (2011)
131. Tavafian, S.S., Jamshidi, A., Mohammad, K., Montazeri, A.: Low back pain education and short term quality of life: a randomized trial. BMC Musculoskelet Disord 8, 21 (2007)
132. Tilbrook, H.E., Cox, H., Hewitt, C.E., Kang'ombe, A.R., Chuang, L.H., Jayakody, S., Aplin, J.D., Semlyen, A., Trewhela, A., Watt, I., Torgerson, D.J.: Yoga for chronic low back pain: a randomized trial. Ann Intern Med 155(9), 569-78 (2011)
133. Tsao, H., Hodges, P.W.: Immediate changes in feedforward postural adjustments following voluntary motor training. Exp Brain Res 181(4), 537-46 (2007)
134. Underwood, M., Mistry, D., Lall, R., Lamb, S.: Predicting response to a cognitive-behavioral approach to treating low back pain: Secondary analysis of the best data set. Arthritis Care Res (Hoboken) 63(9), 1271-9 (2011)
135. Vasseljen, O., Fladmark, A.M.: Abdominal muscle contraction thickness and function after specific and general exercises: a randomized controlled trial in chronic low back pain patients. Man Ther 15(5), 482-9 (2010)
136. Warming, S., Ebbehoj, N.E., Wiese, N., Larsen, L.H., Duckert, J., Tonnesen, H.: Little effect of transfer technique instruction and physical fitness training in reducing low back pain among nurses: a cluster randomised intervention study. Ergonomics 51(10), 1530-48 (2008)
137. Wetherell JL1, Afari N, Rutledge T, Sorrell JT, Stoddard JA, Petkus AJ, Solomon BC, Lehman DH, Liu L, Lang AJ, Atkinson JH. A randomized, controlled trial of acceptance and commitment therapy and cognitive-behavioral therapy for chronic pain. Pain. 2011 Sep;152(9):2098-107.
138. Wheeler, W.J., Gever, L.N.: Functional status of patients with acute low back pain following treatment with carisoprodol 250-mg tablets assessed by the roland-morris disability questionnaire (rmdq). Pain Medicine 11(2), 305 (2010)
139. Whitehurst, D.G., Lewis, M., Yao, G.L., Bryan, S., Raftery, J.P., Mullis, R., Hay, E.M.: A brief pain management program compared with physical therapy for low back pain: results from an economic analysis alongside a randomized clinical trial. Arthritis Rheum 57(3), 466-73 (2007)
140. Wilson-MacDonald, J., Fairbank, J., Frost, H., Yu, L.M., Barker, K., Collins, R., Campbell, H.: The mrc spine stabilization trial: surgical methods, outcomes, costs, and complications of surgical stabilization. Spine (Phila Pa 1976) 33(21), 2334-40 (2008)
141. Worth, S.G.A., Henry, S.M., Bunn, J.Y.: Real-time ultrasound feedback and abdominal hollowing exercises for people with low back pain. NZ J Physiotherapy 35(1), 4-11 (2007)
142. Yakhno, N., Guekht, A., Skoromets, A., Spirin, N., Strachunskaya, E., Ternavsky, A., Olsen, K.J., Moller, P.L.: Analgesic efficacy and safety of lornoxicam quick-release formulation compared with diclofenac potassium: randomised, double-blind trial in acute low back pain. Clin Drug Investig 26(5), 267-77 (2006)
143. Zaina, F., Vismara, L., Menegoni, F., Galli, M., Negrini, S., Villa, V.: Clinical and kinematic evaluation of osteopathy vs specific exercises in obese non-specific chronic low back pain females patients: A randomized controlled trial. Spine Oct 2010, 244 (2010)
144. Ackerman, W.E., Ahmad, M.: Pain relief with intraarticular or medial branch nerve blocks in patients with positive lumbar facet joint spect imaging: A 12-week outcome study. Southern Medical Journal 101(9), 931-934 (2008)
145. Adamczyk, A., Kiebzak, W., Wilk-Franczuk, M., Sliwinski, Z.: Effectiveness of holistic physiotherapy for low back pain. Ortop Traumatol Rehabil 11(6), 562-76 (2009)
146. Ahmed, M.S., Shakoor, M.A., Khan, A.A.: Evaluation of the effects of shortwave diathermy in patients with chronic low back pain. Bangladesh Med Res Counc Bull 35(1), 18-20 (2009)
147. Akbari, A., Khorashadizadeh, S., Abdi, G.: The effect of motor control exercise versus general exercise on lumbar local stabilizing muscles thickness: Randomized controlled trial of patients with chronic low back pain. Journal of Back and Musculoskeletal Rehabilitation 21(2), 105-112 (2008)
148. Albaladejo, C., Kovacs, F.M., Royuela, A., del Pino, R., Zamora, J.: The efficacy of a short education program and a short physiotherapy program for treating low back pain in primary care: a cluster randomized trial. Spine (Phila Pa 1976) 35(5), 483-96 (2010)
149. Anema, J.R., Steenstra, I.A., Bongers, P.M., de Vet, H.C., Knol, D.L., Loisel, P., van Mechelen, W.: Multidisciplinary rehabilitation for subacute low back pain: graded activity or workplace intervention or both? a randomized controlled trial. Spine (Phila Pa 1976) 32(3), 291-8 (2007)
150. Attanayake, A.M.P., Somarathna, K., Vyas, G.H., Dash, S.C.: Clinical evaluation of selected yogic procedures in individuals with low back pain. Ayu 31(2), 245-50 (2010)
151. Becker, A., Leonhardt, C., Kochen, M.M., Keller, S., Wegscheider, K., Baum, E., Donner-Banzhoff, N., Pfingsten, M., Hildebrandt, J., Basler, H.D., Chenot, J.F.: Effects of two guideline implementation strategies on patient outcomes in primary care: a cluster randomized controlled trial. Spine (Phila Pa 1976) 33(5), 473-80 (2008)
152. Bello, A.I., Kalu, N.H., Adegoke, B.O.A., Agyepong-Badu, S.: Hydrotherapy versus land-based exercises in the management of chronic low back pain: A comparative study. Journal of Musculoskeletal Research 13(4), 159-165 (2010)
153. Ben Salah Frih, Z., Fendri, Y., Jellad, A., Boudoukhane, S., Rejeb, N.: Efficacy and treatment compliance of a home-based rehabilitation programme for chronic low back pain: a randomized, controlled study. Ann Phys Rehabil Med 52(6), 485-96 (2009)
154. Bialosky, J.E., Bishop, M.D., Robinson, M.E., Zeppieri, J. G., George, S.Z.: Spinal manipulative therapy has an immediate effect on thermal pain sensitivity in people with low back pain: a randomized controlled trial. Phys Ther 89(12), 1292-303 (2009)
155. Bicalho, E., Palma Setti, J.A., Macagnan, J., Rivas Cano, J.L., Manffra, E.F.: Immediate effects of a high-velocity spine manipulation in paraspinal muscles activity of nonspecifc chronic low-back pain subjects. Manual Therapy 15(5), 469-475 (2010)
156. Birkenmaier, C., Veihelmann, A., Trouillier, H.H., Hausdorf, J., von Schulze Pellengahr, C.: Medial branch blocks versus pericapsular blocks in selecting patients for percutaneous cryodenervation of lumbar facet joints. Reg Anesth Pain Med 32(1), 27-33 (2007)
157. Bishop, P.B., Quon, J.A., Fisher, C.G., Dvorak, M.F.: The chiropractic hospital-based interventions research outcomes (chiro) study: a randomized controlled trial on the effectiveness of clinical practice guidelines in the medical and chiropractic management of patients with acute mechanical low back pain. Spine J 10(12), 1055-64 (2010)
158. Bogefeldt, J., Grunnesjo, M.I., Svardsudd, K., Blomberg, S.: Sick leave reductions from a comprehensive manual therapy programme for low back pain: the gotland low back pain study. Clin Rehabil 22(6), 529-41 (2008)
159. Brennan, G.P., Fritz, J.M., Hunter, S.J., Thackeray, A., Delitto, A., Erhard, R.E.: Identifying subgroups of patients with acute/subacute "nonspecifc" low back pain: results of a randomized clinical trial. Spine 31(6), 623-31 (2006)
160. Brinkhaus, B., Witt, C.M., Jena, S., Linde, K., Streng, A., Wagenpfeil, S., Irnich, D., Walther, H.U., Melchart, D., Willich, S.N.: Acupuncture in patients with chronic low back pain: a randomized controlled trial. Archives of internal medicine. 166(4), 450-7 (2006)
161. Bronfort, G., Maiers, M.J., Evans, R.L., Schulz, C.A., Bracha, Y., Svendsen, K.H., Grimm Jr, R.H., Owens Jr, E.F., Garvey, T.A., Transfeldt, E.E.: Supervised exercise, spinal manipulation, and home exercise for chronic low back pain: A randomized clinical trial. Spine Journal 11(7), 585-598 (2011)
162. Browder, D.A., Childs, J.D., Cleland, J.A., Fritz, J.M.: Effectiveness of an extension-oriented treatment approach in a subgroup of subjects with low back pain: a randomized clinical trial. Phys Ther 87(12), 1608-1815779 (2007)
163. Cairns, M.C., Foster, N.E., Wright, C.: Randomized controlled trial of specific spinal stabilization exercises and conventional physiotherapy for recurrent low back pain. Spine 31(19), 670-81 (2006)
164. Calmels, P., Queneau, P., Hamonet, C., Le Pen, C., Maurel, F., Lerouvreur, C., Thoumie, P.: Effectiveness of a lumbar belt in subacute low back pain: an open, multicentric, and randomized clinical study. Spine (Phila Pa 1976) 34(3), 215-20 (2009)
165. Cecchi, F., Molino-Lova, R., Chiti, M., Pasquini, G., Paperini, A., Conti, A.A., Macchi, C.: Spinal manipulation compared with back school and with individually delivered physiotherapy for the treatment of chronic low back pain: a randomized trial with one-year follow-up. Clin Rehabil 24(1), 26-36 (2010)
166. Chan, C.W., Mok, N.W., Yeung, E.W.: Aerobic exercise training in addition to conventional physiotherapy for chronic low back pain: a randomized controlled trial. Arch Phys Med Rehabil 92(10), 1681-5 (2011)
167. Chang, S.T., Chen, L.C., Chang, C.C., Chu, H.Y., Tsai, K.C.: Effects of piroxicam-beta-cyclodextrin sachets on abnormal postural sway in patients with chronic low back pain. J Clin Pharm Ther 33(5), 495-506 (2008)
168. Chatzitheodorou, D., Mavromoustakos, S., Milioti, S.: The effect of exercise on adrenocortical responsiveness of patients with chronic low back pain, controlled for psychological strain. Clin Rehabil 22(4), 319-28 (2008)
169. Cherkin, D.C., Sherman, K.J., Avins, A.L., Erro, J.H., Ichikawa, L., Barlow, W.E., Delaney, K., Hawkes, R., Hamilton, L., Pressman, A., Khalsa, P.S., Deyo, R.A.: A randomized trial comparing acupuncture, simulated acupuncture, and usual care for chronic low back pain. Arch Intern Med 169(9), 858-66 (2009)
170. Cherkin, D.C., Sherman, K.J., Kahn, J., Wellman, R., Cook, A.J., Johnson, E., Erro, J., Delaney, K., Deyo, R.A.: A comparison of the effects of 2 types of massage and usual care on chronic low back pain: a randomized, controlled trial. Ann Intern Med 155(1), 1{9 (2011)
171. Chiu, C.K., Low, T.H., Tey, Y.S., Singh, V.A., Shong, H.K.: The efficacy and safety of intramuscular injections of methylcobalamin in patients with chronic nonspecific low back pain: a randomised controlled trial. Singapore Med J 52(12), 868-73 (2011)
172. Chown, M., Whittamore, L., Rush, M., Allan, S., Stott, D., Archer, M.: A prospective study of patients with chronic back pain randomised to group exercise, physiotherapy or osteopathy. Physiotherapy 94(1), 21-28 (2008)
173. Cleland, J.A., Fritz, J.M., Kulig, K., Davenport, T.E., Eberhart, S., Magel, J., Childs, J.D.: Comparison of the effectiveness of three manual physical therapy techniques in a subgroup of patients with low back pain who satisfy a clinical prediction rule: a randomized clinical trial. Spine (Phila Pa 1976) 34(25), 2720-9 (2009)
174. Costantino, C., Marangio, E., Coruzzi, G.: Mesotherapy versus systemic therapy in the treatment of acute low back pain: A randomized trial. Evid Based Complement Alternat Med 2011 (2011)
175. Costa, L.O., Maher, C.G., Latimer, J., Hodges, P.W., Herbert, R.D., Refshauge, K.M., McAuley, J.H., Jennings, M.D.: Motor control exercise for chronic low back pain: a randomized placebo-controlled trial. Phys Ther 89(12), 1275-86 (2009)
176. Critchley, D.J., Ratcliffe, J., Noonan, S., Jones, R.H., Hurley, M.V.: Effectiveness and cost-effectiveness of three types of physiotherapy used to reduce chronic low back pain disability: a pragmatic randomized trial with economic evaluation. Spine (Phila Pa 1976) 32(14), 1474-81 (2007)
177. Cuesta-Vargas, A.I., Garcia-Romero, J.C., Arroyo-Morales, M., Diego-Acosta, A.M., Daly, D.J.: Exercise, manual therapy, and education with or without high-intensity deep-water running for nonspecific chronic low back pain: a pragmatic randomized controlled trial. Am J Phys Med Rehabil 90(7), 526-345358 (2011)
178. da Fonseca, J.L., Magini, M., de Freitas, T.H.: Laboratory gait analysis in patients with low back pain before and after a pilates intervention. J Sport Rehabil 18(2), 269-82 (2009)
179. da Silva, A.G., de Sousa, C.P., Koehler, J., Fontana, J., Christo, A.G., Guedes-Bruni, R.R.: Evaluation of an extract of brazilian arnica (solidago chilensis meyen, asteraceae) in treating lumbago. Phytother Res 24(2), 283-7 (2010)
180. Demirel, R., Ucok, K., Kavuncu, V., Gecici, O., Evcik, D., Dundar, U., Solak, O., Mollaoglu, H.: Effects of balneotherapy with exercise in patients with low back pain. J Back Musculoskelet Rehabil 21(4), 263-272 (2008)
181. Demoulin, C., Grosdent, S., Capron, L., Tomasella, M., Somville, P.R., Crielaard, J.M., Vanderthommen, M.:Effectiveness of a semi-intensive multidisciplinary outpatient rehabilitation program in chronic low back pain. Joint Bone Spine 77(1), 58-63 (2010)
182. Di Cesare, A., Giombini, A., Di Cesare, M., Ripani, M., Vulpiani, M.C., Saraceni, V.M.: Comparison between the effects of trigger point mesotherapy versus acupuncture points mesotherapy in the treatment of chronic low back pain: a short term randomized controlled trial. Complement Ther Med 19(1), 19-26 (2011)
183. Diaz Arribas, M.J., Ramos Sanchez, M., Pardo Hervas, P., Lopez Chicharro, J., Angulo Carrere, T., Ortega Molina, P., Astasio Arbiza, P.: E
     ectiveness of the physical therapy godelive denys-struyf method for nonspecific low back pain: primary care randomized control trial. Spine (Phila Pa 1976) 34(15), 1529-38 (2009)
184. Djavid, G.E., Mehrdad, R., Ghasemi, M., Hasan-Zadeh, H., Sotoodeh-Manesh, A., Pouryaghoub, G.: In chronic low back pain, low level laser therapy combined with exercise is more beneficial than exercise alone in the long term: a randomised trial. Aust J Physiother 53(3), 155-60 (2007)
185. Donzelli, S., Domenica, E., Cova, A.M., Galletti, R., Giunta, N.: Two different techniques in the rehabilitation treatment of low back pain: a randomized controlled trial. Eura 42(3), 205-10 (2006)
186. Dufour, N., Thamsborg, G., Oefeldt, A., Lundsgaard, C., Stender, S.: Treatment of chronic low back pain: a randomized, clinical trial comparing group-based multidisciplinary biopsychosocial rehabilitation and intensive individual therapist-assisted back muscle strengthening exercises. Spine (Phila Pa 1976) 35(5), 469-76 (2010)
187. Dundar, U., Solak, O., Yigit, I., Evcik, D., Kavuncu, V.: Clinical effectiveness of aquatic exercise to treat chronic low back pain: a randomized controlled trial. Spine (Phila Pa 1976) 34(14), 1436-40 (2009)
188. Durmus, D., Akyol, Y., Alayli, G., Tander, B., Zahiroglu, Y., Canturk, F.: Effects of electrical stimulation program on trunk muscle strength, functional capacity, quality of life, and depression in the patients with low back pain: a randomized controlled trial. Rheumatol Int 29(8), 947-54 (2009)
189. Durmus, D., Durmaz, Y., Canturk, F.: Effects of therapeutic ultrasound and electrical stimulation program on pain, trunk muscle strength, disability, walking performance, quality of life, and depression in patients with low back pain: a randomized-controlled trial. Rheumatol Int 30(7), 901-10 (2010)
190. Durmus, D., Akyol, Y., Cengiz, K., Terzi, T., Canturk, F.: Effects of therapeutic ultrasound on pain, disability, walking performance, quality of life, and depression in patients with chronic low back pain: A randomized, placebo controlled trial. Turkish Journal of Rheumatology 25(2), 82-87 (2010)
191. Eisenberg, D.M., Post, D.E., Davis, R.B., Connelly, M.T., Legedza, A.T., Hrbek, A.L., Prosser, L.A., Buring, J.E., Inui, T.S., Cherkin, D.C.: Addition of choice of complementary therapies to usual care for acute low back pain: a randomized controlled trial. Spine (Phila Pa 1976) 32(2), 151-8 (2007)
192. Engbert, K., Weber, M.: The effects of therapeutic climbing in patients with chronic low back pain: a randomized controlled study. Spine (Phila Pa 1976) 36(11), 842-9 (2011)
193. Ergun, H., Polat, O., Demirkan, N.A., Gunalp, M., Gurler, S.: The efficacy, safety, and pharmacokinetics of intramuscular and oral phenyramidol in patients with low back pain in an emergency department. Turkish Journal of Medical Sciences 40(1), 71-76 (2010)
194. Ewert, T., Limm, H., Wessels, T., Rackwitz, B., von Garnier, K., Freumuth, R., Stucki, G.: The comparative effectiveness of a multimodal program versus exercise alone for the secondary prevention of chronic low back pain and disability. PM R 1(9), 798-808 (2009)
195. Facci, L.M., Nowotny, J.P., Tormem, F., Trevisani, V.F.: Effects of transcutaneous electrical nerve stimulation (tens) and interferential currents (ifc) in patients with nonspecific chronic low back pain: randomized clinical trial. Sao Paulo Med J 129(4), 206-16 (2011)
196. Farhadi, K., Schwebel, D.C., Saeb, M., Choubsaz, M., Mohammadi, R., Ahmadi, A.: The effectiveness ofwet-cupping for nonspecific low back pain in iran: a randomized controlled trial. Complement Ther Med 17(1), 9-15 (2009)
197. Ferreira, M.L., Ferreira, P.H., Latimer, J., Herbert, R.D., Hodges, P.W., Jennings, M.D., Maher, C.G., Refshauge, K.M.: Comparison of general exercise, motor control exercise and spinal manipulative therapy for chronic low back pain: A randomized trial. Pain 131(1-2), 31-7 (2007)
198. Ferreira, M.L., Ferreira, P.H., Latimer, J., Herbert, R.D., Maher, C., Refshauge, K.: Relationship between spinal stiffness and outcome in patients with chronic low back pain. Man Ther 14(1), 61-7 (2009)
199. Field, T., Hernandez-Reif, M., Diego, M., Fraser, M.: Lower back pain and sleep disturbance are reduced following massage therapy. Journal of Bodywork and Movement Therapies 11(2), 141-145 (2007)
200. Fiore, P., Panza, F., Cassatella, G., Russo, A., Frisardi, V., Solfrizzi, V., Ranieri, M., Di Teo, L., Santamato, A.: Short-term effects of high-intensity laser therapy versus ultrasound therapy in the treatment of low back pain: a randomized controlled trial. Eur J Phys Rehabil Med 47(3), 367-73 (2011)
201. Franca, F.R., Burke, T.N., Hanada, E.S., Marques, A.P.: Segmental stabilization and muscular strengthening in chronic low back pain: a comparative study. Clinics (Sao Paulo) 65(10), 1013-7 (2010)
202. Friedrich, M., Gittler, G.: Long-term effects of a combined exercise and motivation program in patients with chronic low back pain: A five-year follow-up. Pain Practice 9, 121 (2009)
203. Fritz, J.M., Lindsay, W., Matheson, J.W., Brennan, G.P., Hunter, S.J., Moffit, S.D., Swalberg, A., Rodriquez, B.: Is there a subgroup of patients with low back pain likely to benefit from mechanical traction? results of a randomized clinical trial and subgrouping analysis. Spine (Phila Pa 1976) 32(26), 793-800 (2007)
204. George, S.Z., Zeppieri, J. G., Cere, A.L., Cere, M.R., Borut, M.S., Hodges, M.J., Reed, D.M., Valencia, C., Robinson, M.E.: A randomized trial of behavioral physical therapy interventions for acute and sub-acute low back pain (nct00373867). Pain 140(1), 145-57 (2008)
205. Gladwell, V., Head, S., Haggar, M., Beneke, R.: Does a program of pilates improve chronic non-specific low back pain? J Sport Rehabil 15(4), 338-350 (2006)
206. Glazov, G., Schattner, P., Lopez, D., Shandley, K.: Laser acupuncture for chronic non-specific low back pain: a controlled clinical trial. Acupunct Med 27(3), 94-100 (2009)
207. Goldby, L.J., Moore, A.P., Doust, J., Trew, M.E.: A randomized controlled trial investigating the efficiency of musculoskeletal physiotherapy on chronic low back disorder. Spine 31(10), 1083-93 (2006)
208. Gohner, W., Schlicht, W.: Preventing chronic back pain: evaluation of a theory-based cognitive-behavioural training programme for patients with subacute back pain. Patient Educ Couns 64(1-3), 87-95 (2006)
209. Haake, M., Muller, H.H., Schade-Brittinger, C., Basler, H.D., Schafer, H., Maier, C., Endres, H.G., Trampisch, H.J., Molsberger, A.: German acupuncture trials (gerac) for chronic low back pain: randomized, multicenter, blinded, parallel-group trial with 3 groups. Arch Intern Med 167(17), 1892-8 (2007)
210. Hagen, E.M., Odelien, K.H., Lie, S.A., Eriksen, H.R.: Adding a physical exercise programme to brief intervention for low back pain patients did not increase return to work. Scand J Public Health 38(7), 731-8 (2010)
211. Hall, A.M., Maher, C.G., Lam, P., Ferreira, M., Latimer, J.: Tai chi exercise for treatment of pain and disability in people with persistent low back pain: a randomized controlled trial. Arthritis Care Res (Hoboken) 63(11), 1576-83 (2011)
212. Hancock, M.J., Maher, C.G., Latimer, J., McLachlan, A.J., Cooper, C.W., Day, R.O., Spindler, M.F., McAuley, J.H.: Assessment of diclofenac or spinal manipulative therapy, or both, in addition to recommended first-line treatment for acute low back pain: a randomised controlled trial. Lancet 370(9599), 1638-43 (2007)
213. Harts, C.C., Helmhout, P.H., de Bie, R.A., Staal, J.B.: A high-intensity lumbar extensor strengthening program is little better than a low-intensity program or a waiting list control group for chronic low back pain: a randomised clinical trial. Aust J Physiother 54(1), 23-31 (2008)
214. Hartvigsen, J., Morso, L., Bendix, T., Manniche, C.: Supervised and non-supervised nordic walking in the treatment of chronic low back pain: a single blind randomized clinical trial. BMC Musculoskelet Disord 11, 30 (2010)
215. Henchoz, Y., de Goumoens, P., Norberg, M., Paillex, R., So, A.K.: Role of physical exercise in low back pain rehabilitation: a randomized controlled trial of a three-month exercise program in patients who have completed multidisciplinary rehabilitation. Spine (Phila Pa 1976) 35(12), 1192-9 (2010)
216. Henchoz, Y., de Goumoens, P., So, A.K., Paillex, R.: Functional multidisciplinary rehabilitation versus outpatient physiotherapy for non specific low back pain: randomized controlled trial. Swiss Med Wkly 140 (2010). doi:http://dx.doi.org/10.4414/smw.2010.13133.10.4414/smw.2010.13133.
217. Heymans, M.W., Anema, J.R., Vet, H.C., Mechelen, W.: Does flexion-distraction help treat chronic low back pain? (2006)
218. Heymans, M.W., Vet, H.C., Bongers, P.M., Knol, D.L., Koes, B.W., Mechelen, W.: The effectiveness of high-intensity versus low-intensity back schools in an occupational setting: a pragmatic randomized controlled trial. Spine 31(10), 1075-82 (2006)
219. Hondras, M.A., Long, C.R., Cao, Y., Rowell, R.M., Meeker, W.C.: A randomized controlled trial comparing 2 types of spinal manipulation and minimal conservative medical care for adults 55 years and older with subacute or chronic low back pain. J Manipulative Physiol Ther 32(5), 330-43 (2009)
220. Hsieh, L.L., Kuo, C.H., Lee, L.H., Yen, A.M., Chien, K.L., Chen, T.H.: Treatment of low back pain by acupressure and physical therapy: randomised controlled trial. BMJ (Clinical research ed.) 332(7543), 696-700 (2006)
221. Iles, R., Taylor, N.F., Davidson, M., O'Halloran, P.: Telephone coaching can increase activity levels for people with non-chronic low back pain: a randomised trial. J Physiother 57(4), 231- 8 (2011)
222. Inoue, M., Kitakoji, H., Ishizaki, N., Tawa, M., Yano, T., Katsumi, Y., Kawakita, K.: Relief of low back pain immediately after acupuncture treatment{a randomised, placebo controlled trial. Acupunct Med 24(3), 103- 8 (2006)
223. Johnson, R.E., Jones, G.T., Wiles, N.J., Chaddock, C., Potter, R.G., Roberts, C., Symmons, D.P., Watson, P.J., Torgerson, D.J., Macfarlane, G.J.: Active exercise, education, and cognitive behavioral therapy for persistent disabling low back pain: a randomized controlled trial. Spine (Phila Pa 1976) 32(15), 1578-85 (2007)
224. Juni, P., Battaglia, M., Nuesch, E., Hammerle, G., Eser, P., van Beers, R., Vils, D., Bernhard, J., Ziswiler, H.R., Dahler, M., Reichenbach, S., Villiger, P.M.: A randomised controlled trial of spinal manipulative therapy in acute low back pain. Ann Rheum Dis 68(9), 1420-7 (2009)
225. Kapitza, K.P., Passie, T., Bernateck, M., Karst, M.: First non-contingent respiratory biofeedback placebo versus contingent biofeedback in patients with chronic low back pain: a randomized, controlled, double-blind trial. Appl Psychophysiol Biofeedback 35(3), 207-17 (2010)
226. Kell, R.T., Asmundson, G.J.: A comparison of two forms of periodized exercise rehabilitation programs in the management of chronic nonspecific low-back pain. J Strength Cond Res 23(2), 513- 23 (2009)
227. Kell, R.T., Risi, A.D., Barden, J.M.: The response of persons with chronic nonspecfic low back pain to three different volumes of periodized musculoskeletal rehabilitation. J Strength Cond Res 25(4), 1052-64 (2011)
228. Kofotolis, N., Kellis, E.: Effects of two 4-week proprioceptive neuromuscular facilitation programs on muscle endurance, exibility, and functional performance in women with chronic low back pain. Physical therapy. 86(7), 1001-12 (2006)
229. Kolda, Do, an, S., Sonel Tur, B., Kurtai, Y., Atay, M.B.: Comparison of three different approaches in the treatment of chronic low back pain 27(7), 873{81 (2008)
230. Kroll, H.R., Kim, D., Danic, M.J., Sankey, S.S., Gariwala, M., Brown, M.: A randomized, double-blind, prospective study comparing the efficacy of continuous versus pulsed radiofrequency in the treatment of lumbar facet syndrome. J Clin Anesth 20(7), 534-7 (2008)
231. Kulisch, A., Bender, T., Nemeth, A., Szekeres, L.: Effect of thermal water and adjunctive electrotherapy on chronic low back pain: a double-blind, randomized, follow-up study. J Rehabil Med 41(1), 73-9 (2009)
232. Kumar, S., Sharma, V.P., Shukla, R., Dev, R.: Comparative efficacy of two multimodal treatments on male and female sub-groups with low back pain (part ii). J Back Musculoskelet Rehabil 23(1), 1-9 (2010)
233. Kumar, S., Negi, M.P., Sharma, V.P., Shukla, R., Dev, R., Mishra, U.K.: Efficacy of two multimodal treatments on physical strength of occupationally subgrouped male with low back pain. J Back Musculoskelet Rehabil 22(3), 179-88 (2009)
234. Kumar, S., Sharma, V.P., Negi, M.P.: Efficcacy of dynamic muscular stabilization techniques (dmst) over conventional techniques in rehabilitation of chronic low back pain. J Strength Cond Res 23(9), 2651-9 (2009)
235. Lalanne, K., Lafond, D., Descarreaux, M.: Modulation of the flexion-relaxation response by spinal manipulative therapy: a control group study. J Manipulative Physiol Ther 32(3), 203-9 (2009)
236. Lamb, S.E., Hansen, Z., Lall, R., Castelnuovo, E., Withers, E.J., Nichols, V., Potter, R., Underwood, M.R.: Group cognitive behavioural treatment for low-back pain in primary care: a randomised controlled trial and cost-effectiveness analysis. Lancet 375(9718), 916-23 (2010)
237. Lambeek, L.C., van Mechelen, W., Knol, D.L., Loisel, P., Anema, J.R.: Randomised controlled trial of integrated care to reduce disability from chronic low back pain in working and private life. BMJ 340, 1035 (2010)
238. Lau, P.M., Chow, D.H., Pope, M.H.: Early physiotherapy intervention in an accident and emergency department reduces pain and improves satisfaction for patients with acute low back pain: a randomised trial. Aust J Physiother 54(4), 243-9 (2008)
239. Leeuw, M., Goossens, M.E., van Breukelen, G.J., de Jong, J.R., Heuts, P.H., Smeets, R.J., Koke, A.J., Vlaeyen, J.W.: Exposure in vivo versus operant graded activity in chronic low back pain patients: results of a randomized controlled trial. Pain 138(1), 192-207 (2008)
240. Lengsfeld, M., Konig, I.R., Schmelter, J., Ziegler, A.: Passive rotary dynamic sitting at the workplace by office-workers with lumbar pain: a randomized multicenter study. Spine Journal 7(5), 531-540 (2007)
241. Leonardt, C., Keller, S., Chenot, J., Luckmann, J., Basler, H., Wegscheider, B., Baum, E., Donner-Banzho, N., Pfngsten, M., Hildebrandt, F., Kochen, M., Becker, A.: Ttm-based motivational counselling does not increase physical activity of low back pain patients in a primary care setting- a cluster-randomized controlled trial. Patient Educ Couns 70, 50-60 (2008)
242. Lewis, C., Souvlis, T., Sterling, M.: Strain-counterstrain therapy combined with exercise is not more effective than exercise alone on pain and disability in people with acute low back pain: a randomised trial. J Physiother 57(2), 91-8 (2011)
243. Limke, J.C., Rainville, J., Pena, E., Childs, L.: Randomized trial comparing the effects of one set vs two sets of resistance exercises for outpatients with chronic low back pain and leg pain. Eur J Phys Rehabil Med 44(4), 399-405 (2008)
244. Lin, M.L., Lin, M.H., Fen, J.J., Lin, W.T., Lin, C.W., Chen, P.Q.: A comparison between pulsed radiofrequency and electro-acupuncture for relieving pain in patients with chronic low back pain. Acupunct Electrother Res 35(3-4), 133-46 (2010)
245. Little, P., Lewith, G., Webley, F., Evans, M., Beattie, A., Middleton, K., Barnett, J., Ballard, K., Oxford, F., Smith, P., Yardley, L., Hollinghurst, S., Sharp, D.: Randomised controlled trial of alexander technique lessons, exercise, and massage (ateam) for chronic and recurrent back pain. BMJ 337, 884 (2008)
246. Machado, L.A., Maher, C.G., Herbert, R.D., Clare, H., McAuley, J.H.: The effectiveness of the mckenzie method in addition to first-line care for acute low back pain: a randomized controlled trial. BMC Med 8, 10 (2010)
247. Mackawan, S., Eungpinichpong, W., Pantumethakul, R., Chatchawan, U., Hunsawong, T., Arayawichanon, P.: Effects of traditional thai massage versus joint mobilization on substance p and pain perception in patients with non-specific low back pain. Journal of Bodywork and Movement Therapies 11(1), 9-16 (2007)
248. Magnussen, L., Strand, L.I., Skouen, J.S., Eriksen, H.R.: Motivating disability pensioners with back pain to return to work - a randomized controlled trial. Journal of Rehabilitation Medicine 39(1), 81-87 (2007)
249. Marshall, P.: Muscle activation changes after exercise rehabilitation for chronic low back pain. Arch Phys Med Rehabil 89(7), 1305-13 (2008)
250. Marshall, P., Murphy, B.: Self-report measures best explain changes in disability compared with physical measures after exercise rehabilitation for chronic low back pain. Spine (Phila Pa 1976) 33(3), 326-38 (2008)
251. Mazza, M., Mazza, O., Pazzaglia, C., Padua, L., Mazza, S.: Escitalopram 20 mg versus duloxetine 60 mg for the treatment of chronic low back pain. Expert Opin Pharmacother 11(7), 1049-52 (2010)
252. Mibielli, M.A., Geller, M., Cohen, J.C., Goldberg, S.G., Cohen, M.T., Nunes, C.P., Oliveira, L.B., da Fonseca, A.S.: Diclofenac plus b vitamins versus diclofenac monotherapy in lumbago: the dolor study. Curr Med Res Opin 25(11), 2589-99 (2009)
253. Mohseni-Bandpei, M.A., Rahmani, N., Behtash, H., Karimloo, M.: The effect of pelvic floor muscle exercise on women with chronic non-specific low back pain. J Bodyw Mov Ther 15(1), 75-81 (2011)
254. Mohseni-Bandpei, M.A., Critchley, J., Staunton, T., Richardson, B.: A prospective randomised controlled trial of spinal manipulation and ultrasound in the treatment of chronic low back pain. Physiotherapy 92(1), 34-42 (2006)
255. Morone, G., Paolucci, T., Alcuri, M.R., Vulpiani, M.C., Matano, A., Bureca, I., Paolucci, S., Saraceni, V.M.: Quality of life improved by multidisciplinary back school program in patients with chronic non-specific low back pain: a single blind randomized controlled trial. Eur J Phys Rehabil Med 47(4), 533-41 (2011)
256. Muehlbacher, M., Nickel, M.K., Kettler, C., Tritt, K., Lahmann, C., Leiberich, P.K., Nickel, C., Krawczyk, J., Mitterlehner, F.O., Rother, W.K., Loew, T.H., Kaplan, P.: Topiramate in treatment of patients with chronic low back pain: a randomized, double-blind, placebo-controlled study. Clin J Pain 22(6), 526-31 (2006)
257. Muller-Schwefe, G.H.H., Uberall, M.A.: Dysport(registered trademark) for the treatment of myofascial back pain: Results from an open-label, phase ii, randomized, multicenter, dose-ranging study. Scandinavian Journal of Pain 2(1), 25-33 (2011)
258. Muthukrishnan, R., Shenoy, S.D., Jaspal, S.S., Nellikunja, S., Fernandes, S.: The differential effects of core stabilization exercise regime and conventional physiotherapy regime on postural control parameters during perturbation in patients with movement and control impairment chronic low back pain. Sports Med Arthrosc Rehabil Ther Technol 2, 13 (2010)
259. Nassif, H., Brosset, N., Guillaume, M., Delore-Milles, E., Taet, M., Buchholz, F., Toussaint, J.F.: Evaluation of a randomized controlled trial in the management of chronic lower back pain in a french automotive industry: An observational study. Archives of Physical Medicine and Rehabilitation 92(12), 1927-19364 (2011)
260. Newcomer, K.L., Vickers Douglas, K.S., Shelerud, R.A., Long, K.H., Crawford, B.: Is a videotape to change beliefs and behaviors superior to a standard videotape in acute low back pain? a randomized controlled trial. Spine J 8(6), 940-7 (2008)
261. Nigg, B.M., Davis, E., Lindsay, D., Emery, C.: The effectiveness of an unstable sandal on low back pain and golf performance. Clin J Sport Med 19(6), 464-70 (2009)
262. Nordeman, L., Nilsson, B., Moller, M., Gunnarsson, R.: Early access to physical therapy treatment for subacute low back pain in primary health care: a prospective randomized clinical trial. Clin J Pain 22(6), 505-11 (2006)
263. Norris, C., Matthews, M.: The role of an integrated back stability program in patients with chronic low back pain. Complement Ther Clin Pract 14(4), 255-63 (2008)
264. Oleske, D.M., Lavender, S.A., Andersson, G.B., Kwasny, M.M.: Are back supports plus education more effective than education alone in promoting recovery from low back pain?: Results from a randomized clinical trial. Spine (Phila Pa 1976) 32(19), 2050-7 (2007)
265. Pach, D., Brinkhaus, B., Roll, S., Wegscheider, K., Icke, K., Willich, S.N., Witt, C.M.: Efficacy of injections with disci/rhus toxicodendron compositum for chronic low back pain - a randomized placebo-controlled trial. PLoS ONE 6(11) (2011)
266. Paatelma, M., Kilpikoski, S., Simonen, R., Heinonen, A., Alen, M., Videman, T.: Orthopaedic manual therapy, mckenzie method or advice only for low back pain in working adults: a randomized controlled trial with one year follow-up. J Rehabil Med 40(10), 858-63 (2008)
267. Paoloni, M., Bernetti, A., Fratocchi, G., Mangone, M., Parrinello, L., Del Pilar Cooper, M., Sesto, L., Di Sante, L., Santilli, V.: Kinesio taping applied to lumbar muscles influences clinical and electromyographic characteristics in chronic low back pain patients. Eur J Phys Rehabil Med 47(2), 237-44 (2011)
268. Paolucci, T., Morone, G., Iosa, M., Fusco, A., Alcuri, R., Matano, A., Bureca, I., Saraceni, V.M., Paolucci, S.: Psychological features and outcomes of the back school treatment in patients with chronic non-specific low back pain.a randomized controlled study. Eur J Phys Rehabil Med 48(2), 245-53 (2011)
269. Perez-Palomares, S., Olivan-Blazquez, B., Magallon-Botaya, R., De-La-Torre-Beldarrain, M.M.L., Gaspar-Calvo, E., Romo-Calvo, L., Garcia-Lazaro, R., Serrano-Aparicio, B.: Percutaneous electrical nerve stimulation versus dry needling: Effectiveness in the treatment of chronic low back pain. Journal of Musculoskeletal Pain 18(1), 23-30 (2010)
270. Pengel, L.H., Refshauge, K.M., Maher, C.G., Nicholas, M.K., Herbert, R.D., McNair, P.: Physiotherapist-directed exercise, advice, or both for subacute low back pain: a randomized trial. Ann Intern Med 146(11), 787-96 (2007)
271. Petersen, T., Larsen, K., Jacobsen, S.: One-year follow-up comparison of the effectiveness of mckenzie treatment and strengthening training for patients with chronic low back pain: outcome and prognostic factors. Spine (Phila Pa 1976) 32(26), 2948-56 (2007)
272. Powers, C.M., Beneck, G.J., Kulig, K., Landel, R.F., Fredericson, M.: Effects of a single session of posterior-to-anterior spinal mobilization and press-up exercise on pain response and lumbar spine extension in people with nonspecific low back pain. Phys Ther 88(4), 485-493 (2008)
273. Rasmussen-Barr, E., Ang, B., Arvidsson, I., Nilsson-Wikmar, L.: Graded exercise for recurrent low-back pain: a randomized, controlled trial with 6-, 12-, and 36-month follow-ups. Spine (Phila Pa 1976) 34(3), 221-8 (2009)
274. Rasmussen, J., Laetgaard, J., Lindecrona, A.L., Qvistgaard, E., Bliddal, H.: Manipulation does not add to the effect of extension exercises in chronic low-back pain (LBP). a randomized, controlled, double blind study. Joint Bone Spine 75(6), 708-13 (2008)
275. Ritvanen, T., Zaproudina, N., Nissen, M., Leinonen, V., Hanninen, O.: Dynamic surface electromyographic responses in chronic low back pain treated by traditional bone setting and conventional physical therapy. J Manipulative Physiol Ther 30(1), 31-7 (2007)
276. Roche-Leboucher, G., Petit-Lemanac'h, A., Bontoux, L., Dubus-Bausiere, V., Parot-Shinkel, E., Fanello, S., Penneau-Fontbonne, D., Fouquet, N., Legrand, E., Roquelaure, Y., Richard, I.: Multidisciplinary intensive functional restoration versus outpatient active physiotherapy in chronic low back pain: a randomized controlled trial. Spine (Phila Pa 1976) 36(26), 2235-42 (2011)
277. Roche, G., Ponthieux, A., Parot-Shinkel, E., Jousset, N., Bontoux, L., Dubus, V., Penneau-Fontbonne, D., Roquelaure, Y., Legrand, E., Colin, D., Richard, I., Fanello, S.: Comparison of a functional restoration program with active individual physical therapy for patients with chronic low back pain: a randomized controlled trial. Arch Phys Med Rehabil 88(10), 1229-35 (2007)
278. Sahin, N., Albayrak, I., Durmus, B., Ugurlu, H.: Effectiveness of back school for treatment of pain and functional disability in patients with chronic low back pain: a randomized controlled trial. J Rehabil Med 43(3), 224-9 (2011)
279. Santaella Da Fonseca Lopes De Sousa, K., Garcia Orfale, A., Mara Meireles, S., Roberto Leite, J., Natour, J.: Assessment of a biofeedback program to treat chronic low back pain. Journal of Musculoskeletal Pain 17(4), 369-377 (2009)
280. Schiltenwolf, M., Buchner, M., Heindl, B., Reumont, J., Muller, A., Eich, W.: Comparison of a biopsychosocial therapy (BT) with a conventional biomedical therapy (mt) of subacute low back pain in the first episode of sick leave: a randomized controlled trial. Eur Spine J 15(7), 1083-92 (2006)
281. Senna, M.K., Machaly, S.A.: Does maintained spinal manipulation therapy for chronic nonspecific low back pain result in better long-term outcome? Spine (Phila Pa 1976) 36(18), 1427-37 (2011)
282. Shankar, N., Thakur, M., Tandon, O.P., Saxena, A.K., Arora, S., Bhattacharya, N.: Autonomic status and pain profile in patients of chronic low back pain and following electro acupuncture therapy: a randomized control trial. Indian J Physiol Pharmacol 55(1), 25-36 (2011)
283. Sherman, K.J., Cherkin, D.C., Wellman, R.D., Cook, A.J., Hawkes, R.J., Delaney, K., Deyo, R.A.: A randomized trial comparing yoga, stretching, and a self-care book for chronic low back pain. Arch Intern Med 171(22), 2019-26 (2011)
284. Shirado, O., Doi, T., Akai, M., Hoshino, Y., Fujino, K., Hayashi, K., Marui, E., Iwaya, T.: Multicenter randomized controlled trial to evaluate the effect of home-based exercise on patients with chronic low back pain: the japan low back pain exercise therapy study. Spine (Phila Pa 1976) 35(17), 811-9 (2010)
285. Skljarevski, V., Ossanna, M., Liu-Seifert, H., Zhang, Q., Chappell, A., Iyengar, S., Detke, M., Backonja, M.: A double-blind, randomized trial of duloxetine versus placebo in the management of chronic low back pain. Eur J Neurol 16(9), 1041-8 (2009)
286. Skljarevski, V., Zhang, S., Chappell, A.S., Detke, M.J., Murray, I., Backonja, M.: Maintenance of effect of duloxetine in patients with chronic low back pain. European Journal of Pain 13, 195 (2009)
287. Skljarevski, V., Zhang, S., Desaiah, D., Alaka, K.J., Palacios, S., Miazgowski, T., Patrick, K.: Duloxetine versus placebo in patients with chronic low back pain: a 12-week, fixed-dose, randomized, double-blind trial. J Pain 11(12), 1282-90 (2010)
288. Skljarevski, V., Zhang, S., Desaiah, D., Palacios, S., Miazgowski, T., Patrick, K.: Efficacy and safety of duloxetine 60 mg once-daily in patients with chronic low back pain. Journal of Pain 11(4), 38 (2010)
289. Smeets, R.J.E.M., Vlaeyen, J.W.S., Hidding, A., Kester, A.D.M., Van Der Heijden, G.J.M.G., Van Geel, A.C.M., Knottnerus, J.A.: Active rehabilitation for chronic low back pain: Cognitive-behavioral, physical, or both? First direct post-treatment results from a randomized controlled trial [ISRCTN22714229]. BMC Musculoskelet Disord 7 (2006)
290. Smeets, R.J.E.M., Vlaeyen, J.W.S., Kester, A.D.M., Knottnerus, J.A.: Reduction of pain catastrophizing mediates the outcome of both physical and cognitive-behavioral treatment in chronic low back pain. Journal of Pain 7(4), 261-271 (2006)
291. Sorensen, P.H., Bendix, T., Manniche, C., Korsholm, L., Lemvigh, D., Indahl, A.: An educational approach based on a non-injury model compared with individual symptom-based physical training in chronic lbp. A pragmatic, randomised trial with a one-year follow-up. BMC Musculoskelet Disord 11, 212 (2010)
292. Steenstra, I.A., Anema, J.R., Bongers, P.M., de Vet, H.C., Knol, D.L., van Mechelen, W.: The effectiveness of graded activity for low back pain in occupational healthcare. Occup Environ Med 63(11), 718-25 (2006)
293. Steenstra, I.A., Anema, J.R., van Tulder, M.W., Bongers, P.M., de Vet, H.C., van Mechelen, W.: Economic evaluation of a multi-stage return to work program for workers on sick-leave due to low back pain. J Occup Rehabil 16(4), 557-78 (2006)
294. Suen, L.K., Wong, T.K., Chung, J.W., Yip, V.Y.: Auriculotherapy on low back pain in the elderly. Complement Ther Clin Pract 13(1), 63-9 (2007)
295. Suni, J., Rinne, M., Natri, A., Statistisian, M.P., Parkkari, J., Alaranta, H.: Control of the lumbar neutral zone decreases low back pain and improves self-evaluated work ability: a 12-month randomized controlled study. Spine 31(18), 611-20 (2006)
296. Szczurko, O., Cooley, K., Busse, J.W., Seely, D., Bernhardt, B., Guyatt, G.H., Zhou, Q., Mills, E.J.: Naturopathic care for chronic low back pain: a randomized trial. PLoS One 2(9), 919 (2007)
297. Tefner, I.K., Nemeth, A., Laszlofi, A., Kis, T., Gyetvai, G., Bender, T.: The effect of spa therapy in chronic low back pain: a randomized controlled, single-blind, follow-up study. Rheumatol Int 32(10), 3163-9 (2011)
298. Tekur, P., Singphow, C., Nagendra, H.R., Raghuram, N.: Effect of short-term intensive yoga program on pain, functional disability and spinal exibility in chronic low back pain: a randomized control study. J Altern Complement Med 14(6), 637-44 (2008)
299. Tekur, P., Chametcha, S., Hongasandra, R.N., Raghuram, N.: Effect of yoga on quality of life of clbp patients: A randomized control study. Int J Yoga 3(1), 10-7 (2010)
300. Thomas, K.J., MacPherson, H., Thorpe, L., Brazier, J., Fitter, M., Campbell, M.J., Roman, M., Walters, S.J., Nicholl, J.: Randomised controlled trial of a short course of traditional acupuncture compared with usual care for persistent non-specific low back pain. BMJ: British Medical Journal 333(7569), 1-6 (2006)
301. Unsgaard-Tondel, M., Fladmark, A.M., Salvesen, O., Vasseljen, O.: Motor control exercises, sling exercises, and general exercises for patients with chronic low back pain: a randomized controlled trial with 1-year follow-up. Phys Ther 90(10), 1426-40 (2010)
302. van der Roer, N., van Tulder, M., Barendse, J., Knol, D., van Mechelen, W., de Vet, H.: Intensive group training protocol versus guideline physiotherapy for patients with chronic low back pain: a randomised controlled trial. Eur Spine J 17(9), 1193-200 (2008)
303. Vasseljen, O., Unsgaard-Tondel, M., Westad, C., Mork, P.J.: Effect of core stability exercises on feedforward activation of deep abdominal muscles in chronic low back pain: A randomized controlled trial. Spine (Phila Pa 1976) 37(13), 1101-8 (2011)
304. Vong, S.K., Cheing, G.L., Chan, F., So, E.M., Chan, C.C.: Motivational enhancement therapy in addition to physical therapy improves motivational factors and treatment outcomes in people with low back pain: a randomized controlled trial. Arch Phys Med Rehabil 92(2), 176-83 (2011)
305. Weiner, D.K., Perera, S., Rudy, T.E., Glick, R.M., Shenoy, S., Delitto, A.: Efficacy of percutaneous electrical nerve stimulation and therapeutic exercise for older adults with chronic low back pain: a randomized controlled trial. Pain 140(2), 344-57 (2008)
306. Whitfill, T., Haggard, R., Bierner, S.M., Pransky, G., Hassett, R.G., Gatchel, R.J.: Early intervention options for acute low back pain patients: a randomized clinical trial with one-year follow-up outcomes. J Occup Rehabil 20(2), 256-63 (2010)
307. Wilkey, A., Gregory, M., Byfield, D., McCarthy, P.W.: A comparison between chiropractic management and pain clinic management for chronic low-back pain in a national health service outpatient clinic. J Altern Complement Med 14(5), 465-73 (2008)
308. Williams, K., Abildso, C., Steinberg, L., Doyle, E., Epstein, B., Smith, D., Hobbs, G., Gross, R., Kelley, G., Cooper, L.: Evaluation of the effectiveness and efficacy of iyengar yoga therapy on chronic low back pain. Spine (Phila Pa 1976) 34(19), 2066-76 (2009)
309. Witt, C.M., Jena, S., Selim, D., Brinkhaus, B., Reinhold, T., Wruck, K., Liecker, B., Linde, K., Wegscheider, K., Willich, S.N.: Pragmatic randomized trial evaluating the clinical and economic effectiveness of acupuncture for chronic low back pain. American Journal of Epidemiology 164(5), 487-496 (2006)
310. Yildirim, Y., Soyunov, S.: Relationship between learning strategies of patients and proper perception of the home exercise program with non-specic low back pain. J Back Musculoskelet Rehabil 23(3), 137-42 (2010)
311. Zaringhalam, J., Manaheji, H., Rastqar, A., Zaringhalam, M.: Reduction of chronic non-specific low back pain: a randomised controlled clinical trial on acupuncture and baclofen. Chin Med 5, 15 (2010)
