# Supplementary material for: Inclusion and exclusion criteria used in non-specific low back pain trials: a review of randomised controlled trials published between 2006 and 2012
Source: BMC Musculoskelet Disord. 2018 Apr 12;19:113. doi: 10.1186/s12891-018-2034-6 (PMC5898037; doi:10.1186/s12891-018-2034-6)
Supplement: Supplementary file 4 — Table S2. Excluded study characteristics. (PDF 300 kb) [file 12891_2018_2034_MOESM4_ESM.pdf]

Table S2: Excluded study characteristics

Lead author	Publication year	Title	Reason for rejection
Anon	2010	Erratum: Efficacy and safety of tapentadol extended release for the management of chronic low back pain: results of a prospective, randomized, double-blind, placebo- and active-controlled Phase III study	Not a LBP RCT
Ansari, N	2006	A randomized, single blind placebo controlled clinical trial on the effect of continuous ultrasound on low back pain	Non-English language
Barker, K	2008	Treatment of chronic back pain by sensory discrimination training. A Phase I RCT of a novel device (fairmed) vs. TENS	Non-inferiority trial
Basler, H	2006	TTM-based counselling in physiotherapy does not contribute to an increase of adherence to activity recommendations in older adults with chronic low back pain--a randomised controlled trial	Mixed sample (including pathology, pregnancy, neck, thoracic)
Belavy, D	2010	Countermeasures against lumbar spine deconditioning in prolonged bed rest: resistive exercise with and without whole body vibration	Mixed sample (including pathology, pregnancy, neck, thoracic)
Bergholdt, K	2008	Better Backs by Better Beds?	Mixed sample (including pathology, pregnancy, neck, thoracic)
Bruehl, S	2007	Trait Anger Expressiveness and Pain-Induced Beta-Endorphin Release: Support for the Opioid Dysfunction Hypothesis	Only objective or psychological outcomes
Bruehl, S	2008	Anger Management Style and Emotional Reactivity to Noxious Stimuli Among Chronic Pain Patients and Healthy Controls: The Role of Endogenous Opioids	Only objective or psychological outcomes
Buttagat, V	2011	The immediate effects of traditional Thai massage on heart rate variability and stress-related parameters in patients with back pain associated with myofascial trigger points	Mixed sample (including pathology, pregnancy, neck, thoracic)
Buynak, R	2009	Dose Stability Of Tapentadol ER For The Relief Of Chronic Low Back Pain: Results Of A Randomized, Active≠ And Placebo≠Controlled Study	Not a LBP RCT
Cabitza, P	2008	Efficacy and safety of eperisone in patients with low back pain: a double blind randomized study	Non-inferiority trial

Lead author	Publication year	Title	Reason for rejection
Casserley-Feeney, S	2007	The ACCESS trial -- randomised controlled trial of public hospital-based versus private clinic-based physiotherapy for low back pain: clinical outcomes	Multiple publication
Cevik, R	2007	Effect of new traction technique of prone position on distraction of lumbar vertebrae and its relation with different application of heating therapy in low back pain	Pilot or prelimianary study
Christiansen, S	2010	A short goal-pursuit intervention to improve physical capacity: A randomized clinical trial in chronic back pain patients	Mixed sample (including pathology, pregnancy, neck, thoracic)
Clark, D	2010	Tolerance and opioid-induced hyperalgesia in clinical populations	Not a LBP RCT
Cleland, J	2009	Comparison of the Effectiveness of Three Manual Physical Therapy Techniques in a Subgroup of Patients With Low Back Pain Who Satisfy a Clinical Prediction Rule A Randomized Clinical Trial	Pilot or prelimianary study
Codding, C	2009	Efficacy and Safety Evaluation of 12 Weeks Extended-Release Hydrocodone/Acetaminophen Treatment in Patients with Chronic Low Back Pain (CLBP) by Prior Opioid Use	Not a LBP RCT
Cohen, S	2008	Lumbar zygapophysial (facet) joint radiofrequency denervation success as a function of pain relief during diagnostic medial branch blocks: a multicenter analysis	Not a LBP RCT
Cox, J	2009	Letter to the Editor: a randomized controlled trial comparing 2 types of spinal manipulation and minimal conservative meical care for adults 55 yeras and older with subacute or chronic low back pain	Not a LBP RCT
Curnow, D	2009	Altered motor control, posture and the Pilates method of exercise prescription	Mixed sample (including pathology, pregnancy, neck, thoracic)
Dagenais, S	2007	Prolotherapy injections for chronic low-back pain (Review)	Not a LBP RCT
Day, I	2008	Can Topical Anesthetic Reduce the Pain Associated with Diagnostic Blocks of the Lumbosacral Spine?	Mixed sample (including pathology, pregnancy, neck, thoracic)
Demoulin, C	2006	Benefits of a Physical Training Program After Back School for Chronic Low Back Pain Patients	Not a LBP RCT

Lead author	Publication year	Title	Reason for rejection
Deshpande, A	2007	Opioids for chronic low-back pain	Not a LBP RCT
Desmoulin, G	2007	Initial results using Khan Kinetic Treatment(trademark) as a low back pain treatment option	Mixed sample (including pathology, pregnancy, neck, thoracic)
Engers, A	2008	Individual patient education for low back pain	Not a LBP RCT
Etropolski, M	2010	Dose Conversion Between Tapentadol Immediate and Extended Release for Low Back Pain	Crossover design
Etropolski, M	2009	A randomized, double-blind, placebo- and active-con- trolled phase III study of tapentadol ER for chronic low back pain: analysis of efficacy endpoint sensitivity	Not a LBP RCT
Evans, D	2010	Characteristics and Predictors of Short-Term Outcomes in Individuals Self-selecting Yoga or Physical Therapy for Treatment of Chronic Low Back Pain	Not a LBP RCT
Gatti, R	2011	Efficacy of trunk balance exercises for individuals with chronic low back pain: a randomized clinical trial	Mixed sample (including pathology, pregnancy, neck, thoracic)
George, S	2011	Brief psychosocial education, not core stabilization, reduced incidence of low back pain: results from the Prevention of Low Back Pain in the Military (POLM) cluster randomized trial	Not a LBP RCT
George, S	2009	Psychosocial education improves low back pain beliefs: results from a cluster randomized clinical trial (NCT00373009) in a primary prevention setting	Not a LBP RCT
George, S	2010	Comparison of Graded Exercise and Graded Exposure Clinical Outcomes for Patients With Chronic Low Back Pain	Not a LBP RCT
Gould, E	2009	The Pain Quality Response Profile of Oxymorphone Extended Release in the Treatment of Low Back Pain	Secondary report of RCT
Guthrie, R	2011	The Effect of Traditional Bridging or Suspension Exercise Bridging on Lateral Abdominal Thickness in Individuals with Low Back Pain	Only objective or psychological outcomes

Lead author	Publication year	Title	Reason for rejection
Hale, M	2007	Efficacy and Safety of OPANA ER (Oxymorphone Extended Release) for Relief of Moderate to Severe Chronic Low Back Pain in Opioid-Experienced Patients: A 12-Week, Randomized, Double-blind, placebo-controlled Study	Mixed sample (including pathology, pregnancy, neck, thoracic)
Hale, M	2010	Once-daily OROS hydromorphone ER compared with placebo in opioid-tolerant patients with chronic low back pain	Mixed sample (including pathology, pregnancy, neck, thoracic)
Hancock, M	2010	Letter	Not a LBP RCT
Hasegawa, T	2009	Acupuncture for acute non≠specific low back pain: a randomized, controlled, placebo trial	Not a LBP RCT
Helmhout, P	2008	Isolated lumbar extensor strengthening versus regular physical therapy in an army working population with nonacute low back pain: a randomized controlled trial	Not a LBP RCT
Helmhout, P	2010	Prognostic factors for perceived recovery or functional improvement in non-specific low back pain: secondary analyses of three randomized clinical trials	Not a LBP RCT
Henchos, Y	2010	Cost-utility analysis of a three-month exercise programme vs usual care following multidisciplinary rehabilitation for chronic low back pain	Not a LBP RCT
Hides, J	2011	Effect of Motor Control Training on Muscle Size and Football Games Missed from Injury	Not a LBP RCT
Hlobil, H	2007	Substantial sick-leave costs savings due to a graded activity intervention for workers with non-specific sub-acute low back pain	Secondary report of RCT
Hollinghurst, S	2008	Randomised controlled trial of Alexander technique lessons, exercise, and massage (ATEAM) for chronic and recurrent back pain: economic evaluation	Secondary report of RCT
Hurley, D	2009	A walking programme and a supervised exercise class versus usual physiotherapy for chronic low back pain: a single-blinded randomised controlled trial. (The Supervised Walking In comparison to Fitness Training for Back Pain (SWIFT) Trial)	Pilot or prelimianary study

Lead author	Publication year	Title	Reason for rejection
Hush, J	2006	TENS of unknown value in the treatment of chronic low back pain	Not a LBP RCT
Ijzelenberg, H	2007	Effectiveness of a back pain prevention program: A cluster randomized controlled trial in an occupational setting	Not a LBP RCT
Ikegami, S	2010	Anti-Nociceptive Effects of Elcatonin Injection for Postmenopausal Women with Back Pain: A Randomized Controlled Trial	Mixed sample (including pathology, pregnancy, neck, thoracic)
Inoue, M	2010	Comparison of the effectiveness of acupuncture treatment and local anasthetic injection for low back pain: a randomized controlled trial	Mixed sample (including pathology, pregnancy, neck, thoracic)
Jans, M	2006	Intermittent follow-up treatment with Cesar exercise therapy in patients with subacute or chronic aspecific low back pain: results of a randomized, controlled trial with a 1.5-year follow-up	Non-English language
Kasis, A	2009	Significantly Improved Outcomes With a Less Invasive Posterior Lumbar Interbody Fusion Incorporating Total Facetectomy	Mixed sample (including pathology, pregnancy, neck, thoracic)
Katz, N	2009	Tanezumab, an Anti-Nerve Growth Factor (NGF) antibody, for the treatment of chronic low back pain (CLBP) - a randomized, controlled, double-blind, phase 2 trial	Not a LBP RCT
Katz, N	2007	A 12-week, randomized, placebo-controlled trial assessing the safety and efficacy of oxymorphone extended release for opioidnaive patients with chronic low back pain	Mixed sample (including pathology, pregnancy, neck, thoracic)
Kavanagh, S	2009	Tapentadol extended release (er) for chronic low back pain: results of euroqol-5 dimension (eq-5d) and short form-36 (sf-36) health status questionnaires	Not a LBP RCT
Kettenmann, B	2007	Impact of Continuous Low Level Heatwrap Therapy in Acute Low Back Pain Patients: Subjective and Objective Measurements	Mixed sample (including pathology, pregnancy, neck, thoracic)
Kool, J	2007	Function-Centered Rehabilitation Increases Work Days in Patients With Nonacute Nonspecific Low Back Pain: 1-Year Results From a Randomized Controlled Trial	Only objective or psychological outcomes

Lead author	Publication year	Title	Reason for rejection
Kovacs, F	2007	A Comparison of Two Short Education Programs for Improving Low Back Pain-Related Disability in the Elderly	Mixed sample (including pathology, pregnancy, neck, thoracic)
Kullich, W	2006	Additional outcome improvement in the rehabilitation of chronic low back pain after nuclear resonance therapy	Mixed sample (including pathology, pregnancy, neck, thoracic)
Kullich, W	2006	The effect of MBST nuclear resonancetherapy with a complex 3-dimensional electromagnetic nuclear resonance field on patients with low back pain	Mixed sample (including pathology, pregnancy, neck, thoracic)
Lamb, S	2010	A multicentred randomised controlled trial of a primary care-based cognitive behavioural programme for low back pain. The Back Skills Training (best) trial	Multiple publication
Lee, J	2010	Therapeutic Trial of Fluoroscopic Interlaminar Epidural Steroid Injection for Axial Low Back Pain: Effectiveness and Outcome Predictors	Not a LBP RCT
Lee, T	2011	Pharmacologic treatment for low back pain: One component of pain care	Not a LBP RCT
Leichtfried, V	2010	Can bright light therapy ameliorate symptoms associated with low back pain (LBP)? A randomized controlled trial	Not a LBP RCT
Lewis, C	2010	A randomised controlled study examining the short-term effects of strainecounterstrain treatment on quantitative sensory measures at digitally tender points in the low back	Crossover design
Li, C	2008	Analgesic efficacy and tolerability of flupirtine vs. Tramadol in patients with subacute low back pain: a double-blind multicentre trial	Non-inferiority trial
Liddle, D	2007	Advice for the management of low back pain: A systematic review of randomised controlled trials	Not a LBP RCT
Long, M	2008	The comparative prognostic value of directional preference and centralization: A useful tool for front-line clinicians?	Secondary report of RCT

Lead author	Publication year	Title	Reason for rejection
Macfarlane, G	2008	Changing patient perceptions of their illness: Can they contribute to an improved outcome for episodes of musculoskeletal pain?	Not a LBP RCT
Magnusson, M	2008	Motor Control Learning in Chronic Low Back Pain	Not a LBP RCT
Mandara, A	2008	A randomised controlled trial on the effectiveness of osteopathic manipulative treatment of chronic low back pain	Not a LBP RCT
Mattila, R	2007	The Effects of Lifestyle Intervention for Hypertension on Low Back Pain	Mixed sample (including pathology, pregnancy, neck, thoracic)
Mehling, W	2006	Breath therapy for chronic low back pain	Not a LBP RCT
Mehta, S	2009	Evaluation of eperisone hydrochloride in the treatment of acute musculoskeletal spasm associated with low back pain: A randomized, double-blind, placebo-controlled trial	Mixed sample (including pathology, pregnancy, neck, thoracic)
Meng, K	2011	Intermediate and Long-term Effects of a Standardized Back School for Inpatient Orthopedic Rehabilitation on Illness Knowledge and Self-management Behaviors	Only objective or psychological outcomes
Mirovsky, Y	2006	The effect of ambulatory lumbar traction combined with treadmill on patients with chronic low back pain	Mixed sample (including pathology, pregnancy, neck, thoracic)
Miyazaki, S	2009	Applicability of Press Needles to a Double-blind Trial A Randomized, Double-blind, Placebo-controlled Trial	Mixed sample (including pathology, pregnancy, neck, thoracic)
Murtezani, A	2011	A comparison of high intensity aerobic exercise and passive modalities for the treatment of workers with chronic low back pain: a randomized, controlled trial	Mixed sample (including pathology, pregnancy, neck, thoracic)

Lead author	Publication year	Title	Reason for rejection
Najm, W	2008	German acupuncture trials (GERAC) for chronic low back pain	Not a LBP RCT
Nath, S	2008	Percutaneous Lumbar Zygapophysial (Facet) Joint Neurotomy Using Radiofrequency Current, in the Management of Chronic Low Back Pain	Mixed sample (including pathology, pregnancy, neck, thoracic)
Nelson-Wong, E	2010	Changes in muscle activation patterns and subjective low back pain ratings during prolonged standing in response to an exercise intervention	Mixed sample (including pathology, pregnancy, neck, thoracic)
North, R	2007	2blackwell publishing inc spinal cord stimulation with interleaved pulses: a randomized, controlled trial	Mixed sample (including pathology, pregnancy, neck, thoracic)
O'Brien, N	2006	Randomised, controlled trial comparing physiotherapy and Pilates in the treatment of ordinary low back pain	Not a LBP RCT
O`Donnel, J	2009	The Effectiveness of a Weak Opioid Medication versus a Cyclo-oxygenase-2 (COX-2) Selective Non-steroidal Anti-inflammatory Drug in Treating Flare-up of Chronic Low-back Pain: Results from Two Randomized, Double-blind, 6-week Studies	Not a LBP RCT
Padua, R	2009	Re (letter): A randomized study of back school in women with chronic low back pain. Quality of life at three, six, and twelve months follow-up.	Not a LBP RCT
Pareek, A	2009	Aceclofenac-tizanidine in the treatment of acute low back pain: a double-blind, double-dummy, randomized, multicentric, comparative study against aceclofenac alone	Mixed sample (including pathology, pregnancy, neck, thoracic)
Peniston, J	2009	Oxymorphone Extended Release for the Treatment of Chronic Low Back Pain: A Retrospective Pooled Analysis of Enriched-Enrollment Clinical Trial Data Stratified According to Age, Sex, and Prior Opioid Use	Not a LBP RCT
Perrot, S	2006	Efficacy and Tolerability of Paracetamol /Tramadol (325 mg/37.5 mg) Combination Treatment Compared with Tramadol (50 mg) Monotherapy in Patients with Subacute Low Back Pain: A Multicenter, Randomized, Double-Blind, Parallel-Group, 10-Day Treatment	Not a LBP RCT

Lead author	Publication year	Title	Reason for rejection
Petersen, T	2011	The mckenzie Method Compared With Manipulation When Used Adjunctive to Information and Advice in Low Back Pain Patients Presenting With Centralization or Peripheralization	Mixed sample (including pathology, pregnancy, neck, thoracic)
Petrofsky, J	2008	Improving the outcomes after back injury by a core muscle strengthening program	Not a LBP RCT
Podichetty, V	2008	Re: Oleske D M, Lavender S A, Andersson G B, et al. Are back supports plus education more effective than education alone in promoting recovery from low back pain? Results from a randomized clinical trial	Not a LBP RCT
Popovic, D	2009	Lumbar Stimulation Belt for Therapy of Low-Back Pain	Mixed sample (including pathology, pregnancy, neck, thoracic)
Portenoy, R	2007	Fentanyl buccal tablet (FBT) for relief of breakthrough pain in opioid-treated patients with chronic low back pain: a randomized, placebo-controlled study	Specific LBP
Pozo-Cruz, B	2011	Effects of whole body vibration therapy on main outcome measures for chronic non-specific low back pain: a singleblind randomized controlled trial	Pilot or preliminary study
Pushpika Attanayake, A	2010	Clinical evaluaion of selected yogic procedures in individuals with low back pain.	Only objective or psychological outcomes
Quartana, P	2007	Attentional strategy moderates effects of pain catastrophizing on symptom-specific physiological responses in chronic low back pain patients	Mixed sample (including pathology, pregnancy, neck, thoracic)
Ralph, L	2009	Improvement in Functional Status with Carisoprodol 250-mg Tablets in Patients with Acute Lower Back Spasm: A Randomized, Double-blind, Placebo-controlled Trial	Not a LBP RCT
Ralph, L	2008	Double-blind, placebo-controlled trial of carisoprodol 250-mg tablets in the treatment of acute lower-back spasm	Mixed sample (including pathology, pregnancy, neck, thoracic)
Rauck, R	2006	A randomized, open-label study of once-a-day AVINZA (morphine sulfate extended-release capsules) versus twice-a-day oxycontin (oxycodone hydrochloride controlled release tablets) for chronic low back pain: the extension phase of the ACTION trial	Mixed sample (including pathology, pregnancy, neck, thoracic)

Lead author	Publication year	Title	Reason for rejection
Rauck, R	2006	The ACTION study: a randomized, open-label, multicenter trial comparing once-a-day extended-release morphine sulfate capsules (AVINZA) to twice-a-day controlled release oxycodone hydrochloride tablets (oxycontin) for the treatment of chronic	Secondary report of RCT
Rivero-Arias, O	2006	Cost-Utility Analysis of Physiotherapy Treatment Compared With Physiotherapy Advice in Low Back Pain	Not a LBP RCT
Romano, C	2009	Pregabalin, celecoxib, and their combination for treatment of chronic low-back pain	Mixed sample (including pathology, pregnancy, neck, thoracic)
Rusinyol, F	2009	Effect of two different doses of eperisone in the treatment of acute low back pain	Mixed sample (including pathology, pregnancy, neck, thoracic)
Ruth, M	2010	Laser acupuncture for chronic back pain. A double-blind clinical study	Non-English language
Schimmel, J	2009	No effect of traction in patients with low back pain: a single centre, single blind, randomized controlled trial of Intervertebral Differential Dynamics Therapy	Mixed sample (including pathology, pregnancy, neck, thoracic)
Schmidt-Wilcke, T	2006	Affective components and intensity of pain correlate with structural differences in gray matter in chronic back pain patients	Not a LBP RCT
Schwarz, I	2007	Relative responsiveness of 3 different types of clinical outcome measures on chiropractic patients with low back pain	Not a LBP RCT
Serfer, G	2010	Randomized, double-blind trial of carisoprodol 250 mg compared with placebo and carisoprodol 350 mg for the treatment of low back spasm	Mixed sample (including pathology, pregnancy, neck, thoracic)
Sertpoyraz, F	2009	Comparison of isokinetic exercise versus standard exercise training in patients with chronic low back pain: a randomized controlled study	Mixed sample (including pathology, pregnancy, neck, thoracic)
Shakoor, A	2010	Evaluation of the effects of selective rehabilitation on the patients with chronic low back pain	Not a LBP RCT
Sherman, K	2010	Treatment Expectations and Preferences as Predictors of Outcome of Acupuncture for Chronic Back Pain	Specific LBP

Lead author	Publication year	Title	Reason for rejection
Sherman, K	2009	Characteristics of patients with chronic back pain who benefit from acupuncture	Secondary report of RCT
Shimoji, K	2007	Pain relief by transcutaneous electric nerve stimulation with bidirectional modulated sine waves in patients with chronic back pain: a randomized, double-blind, sham-controlled study	Mixed sample (including pathology, pregnancy, neck, thoracic)
Shum, G	2006	Movement coordination of the lumbar spine and hip during a picking up activity in low back pain subjects	Not a LBP RCT
Skljarevski, V	2010	Efficacy and safety of duloxetine 60 mg once-daily in pa- tients with chronic low back pain	Mixed sample (including pathology, pregnancy, neck, thoracic)
Skljarevski, V	2010	Effect of Duloxetine 60mg Once Daily Versus Placebo in Patients with Chronic Low Back Pain: A 12-Week, Randomized, Double-Blind Trial	Not a LBP RCT
Slater, M	2009	Preventing Progression to Chronicity in First Onset, Subacute Low Back Pain: An Exploratory Study	Mixed sample (including pathology, pregnancy, neck, thoracic)
Smeets, R	2009	Chronic low back pain: Physical training, graded activity with problem solving training, or both? The one-year post-treatment results of a randomized controlled trial	Not a LBP RCT
Smeets, R	2006	Do lumbar stabilising exercises reduce pain and disability in patients with recurrent low back pain?	Not a LBP RCT
Smeets, R	2009	Treatment expectancy and credibility are associated with the outcome of both physical and cognitive-behavioral treatment in chronic low back pain	Not a LBP RCT
Smith, A	2007	The effect of the Feldenkrais method on pain and anxiety in people experiencing chronic low back pain	Pilot or prelimianary study
Sokunbi, O	2007	Changes in plasma concentration of serotonin in response to spinal stabilisation exercises in chronic low back pain patient	Only objective or psychological outc
Soonawalla, D	2008	Efficacy of thiocolchicoside in Indian patients suffering from low back pain associated with muscle spasm	Mixed sample (including pathology, pregnancy, neck, thoracic)

Lead author	Publication year	Title	Reason for rejection
Steiner, D	2011	Efficacy and Safety of the Seven-Day Buprenorphine Transdermal System in Opioid-Naeive Patients with Moderate to Severe Chronic Low Back Pain: An Enriched, Randomized, Double-Blind, Placebo-Controlled Study	Specific LBP
Steiner, D	2011	Efficacy and Safety of Buprenorphine Transdermal System (BTDS) for Chronic Moderate to Severe Low Back Pain: A Randomized, Double-Blind Study	Specific LBP
Steiner, D	2009	The efficacy and safety of buprenorphine transdermal system (BTDS) in subjects with moderate to severe low back pain: A double-blind study	Mixed sample (including pathology, pregnancy, neck, thoracic)
Sutlive, T	2009	Comparison of short-term response to two spinal manilpulation techniques for patients with low back pain in a military beneficiary population	Mixed sample (including pathology, pregnancy, neck, thoracic)
Tavafian, S	2008	A Randomized Study of Back School in Women With Chronic Low Back Pain	Mixed sample (including pathology, pregnancy, neck, thoracic)
Tavafian, S	2011	Treatment of Chronic Low Back Pain A Randomized Clinical Trial Comparing Multidisciplinary Group-based Rehabilitation Program and Oral Drug Treatment With Oral Drug Treatment Alone	Specific LBP
Tavafian, S	2007	Low back pain education and short term quality of life: a randomized trial	Mixed sample (including pathology, pregnancy, neck, thoracic)
Tilbrook, H	2011	Yoga for Chronic Low Back Pain	Mixed sample (including pathology, pregnancy, neck, thoracic)
Tsao, H	2007	Immediate changes in feedforward postural adjustments following voluntary motor training	Only objective or psychological outcomes
Underwood, M	2011	Predicting Response to a Cognitive-Behavioral Approach to Treating Low Back Pain: Secondary Analysis of the best Data Set	Not a LBP RCT
Vasseljen, O	2010	Abdominal muscle contraction thickness and function after specific and general exercises: A randomized controlled trial in chronic low back pain patients	Only objective or psychological outcomes

Lead author	Publication year	Title	Reason for rejection
Warming, S	2008	Little effect of transfer technique instruction and physical fitness training in reducing low back pain among nurses: a cluster randomised intervention study	Not a LBP RCT
Wetherell, J	2011	A randomized controlled trial of acceptance and commitment therapy and cognitive behavioural therapy for chronic pain	Specific LBP
Westrom, K	2010	Individualized chiropractic and integrative care for low back pain: the design of a randomized clinical trial using a mixed-methods approach	Pilot or prelimianary study
Wheeler, W	2010	Functional status of patients with acute low back pain following treatment with carisoprodol 250-mg tablets assessed by the roland-morris disability questionnaire (RMDQ)	Not a LBP RCT
Whitehurst, D	2007	A Brief Pain Management Program Compared With Physical Therapy for Low Back Pain: Results From an Economic Analysis Alongside a Randomized Clinical Trial	Secondary report of RCT
Wilson-MacDonald, J	2008	The MRC spine stabilization trial: surgical methods, outcomes, costs, and complications of surgical stabilization	Mixed sample (including pathology, pregnancy, neck, thoracic)
Worth, S	2007	Real-time ultrasound feedback and abdominal hollowing exercises for people with low back pain	Only objective or psychological outcomes
Yakhno, N	2006	Analgesic Efficacy and Safety of Lornoxicam Quick-Release Formulation Compared with Diclofenac Potassium Randomised, Double-Blind Trial in Acute Low Back Pain	Non-inferiority trial
Zaina, F	2010	Clinical and kinematic evaluation of osteopathy vs specific exercises in obese non-specific chronic low back pain females patients: a randomized controlled trial	Not a LBP RCT
